# Constructing Donor-Resonance-Donor Molecules for Acceptor-Free Bipolar Organic Semiconductors

**DOI:** 10.34133/2021/9525802

**Published:** 2021-02-05

**Authors:** He Jiang, Jibiao Jin, Zijie Wang, Wuji Wang, Runfeng Chen, Ye Tao, Qin Xue, Chao Zheng, Guohua Xie, Wei Huang

**Affiliations:** ^1^ Key Laboratory for Organic Electronics and Information Displays & Jiangsu Key Laboratory for Biosensors, Institute of Advanced Materials (IAM), Nanjing University of Posts & Telecommunications, 9 Wenyuan Road, Nanjing 210023, China; ^2^ Department of Physical Science and Technology, Central China Normal University, Wuhan 430079, China; ^3^ Sauvage Center for Molecular Sciences, Hubei Key Lab on Organic and Polymeric Optoelectronic Materials, Department of Chemistry, Wuhan University, Wuhan 430072, China; ^4^ Frontiers Science Center for Flexible Electronics (FSCFE), Shaanxi Institute of Flexible Electronics (SIFE) & Shaanxi Institute of Biomedical Materials and Engineering (SIBME), Northwestern Polytechnical University (NPU), 127 West Youyi Road, Xi'an 710072, China

## Abstract

Organic semiconductors with bipolar transporting character are highly attractive as they offer the possibility to achieve high optoelectronic performance in simple device structures. However, the continual efforts in preparing bipolar materials are focusing on donor-acceptor (D-A) architectures by introducing both electron-donating and electron-withdrawing units into one molecule in static molecular design principles. Here, we report a dynamic approach to construct bipolar materials using only electron-donating carbazoles connected by N-P=X resonance linkages in a donor-resonance-donor (D-r-D) structure. By facilitating the stimuli-responsive resonance variation, these D-r-D molecules exhibit extraordinary bipolar properties by positively charging one donor of carbazole in enantiotropic N^+^=P-X^-^ canonical forms for electron transport without the involvement of any acceptors. With thus realized efficient and balanced charge transport, blue and deep-blue phosphorescent organic light emitting diodes hosted by these D-r-D molecules show high external quantum efficiencies up to 16.2% and 18.3% in vacuum-deposited and spin-coated devices, respectively. These results *via* the D-r-D molecular design strategy represent an important concept advance in constructing bipolar organic optoelectronic semiconductors dynamically for high-performance device applications.

## 1. Introduction

**Figure 1:**
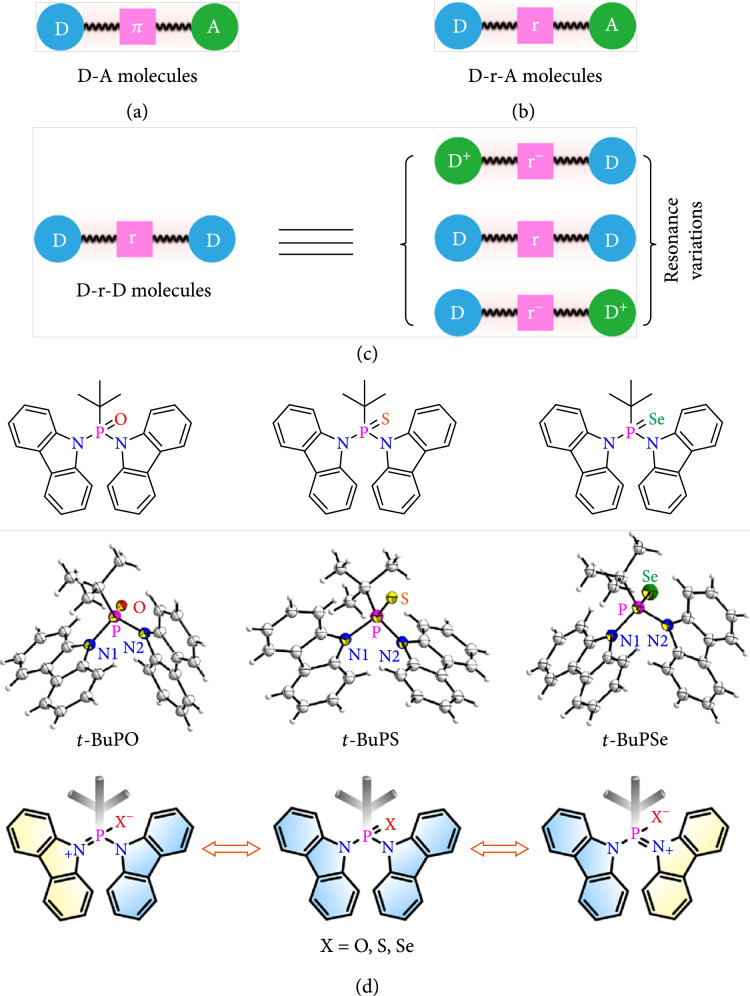
Molecular design of the D-r-D bipolar materials. (a) D-A, (b) D-r-A, and (c) D-r-D architectures of bipolar molecules. (d) Structural formulas, single-crystal structures, and main resonance variations of the D-r-D molecules.

Organic semiconductors with bipolar transporting character are of key importance in organic electronics to achieve the balanced hole and electron transportation for high-performance device applications, including organic light emitting diodes (OLEDs) [1–3], organic solar cells (OSCs) [4, 5], organic field effect transistors (OFETs) [6, 7], photodetectors [8], memory devices [9], and organic afterglow applications [10, 11]. A commonly adopted molecular design strategy for bipolar organic semiconductors is to construct donor-acceptor (D-A) molecular skeletons with electron-donating and electron-withdrawing moieties arranged into one molecule for electron and hole transport, respectively (Figure 1(a)) [12, 13]. The electronic properties of thus designed D-A molecules are readily modulated on the basis of the inherent relationship between molecules and donor (D)/acceptor (A) groups [14]. However, compared to the individual D and A components, D-A molecules commonly show narrower bandgaps ($E_{g}\text{s}$) with significant bathochromic shift in emission and lower triplet energies ($E_{T}\text{s}$) with much extended *π*-conjugation owing to the inevitable intramolecular charge transfer (ICT) interactions between D and A moieties [15]. These intrinsic features of D-A molecules limit significantly their development in high-performance blue emitting molecules with large $E_{g}$ or host materials with high $E_{T}$ for blue/deep-blue phosphorescent OLEDs (PhOLEDs) [12, 16].

Compared to the direct and straightforward static D-A strategy in achieving bipolar characteristics, we found recently that organic resonance molecules can dynamically change the widely recognized donor unit of carbazole to be electron transportable upon resonance variation [17]. By directly linking arylamine and phenylphosphine oxide (sulfide or selenide) moieties in N-P = X (X = O, S, or Se) resonance structures, dynamically bipolar organic semiconductors were constructed by the resonance variation-based dynamic adaptation (RVDA) strategy [18]. The resonance charge redistribution to generate various neutral N-P = X and polarized N^+^ = P-X^-^ canonical forms dynamically can tune the electronic properties *in situ* upon environmental stimuli, resulting in stimuli-responsive behaviors (Figure 1(b)). Moreover, in virtue of the insulating resonance linkage, *π*-conjugation can be well controlled without reducing much of the excited energy. These resonance molecules designed in N-P=O and N-P=S resonance (r) linked D-A architecture (D-r-A) are excellent bipolar host materials of blue PhOLEDs, showing the selectively and remarkably enhanced electronic properties for high device performances. It should be noted that the resonance linkage can dynamically and selectively tune the electronic properties, while produce no negative influences on photophysical properties of the organic semiconductors. More importantly, the resonance linkage can be also served as the valve of dynamic response by gating the intramolecular charge transfer during the resonance isomerization.

Here, on the basis of RVDA strategy, we present a donor-resonance-donor (D-r-D) molecular design approach that operates *via* resonance variation to construct bipolar materials dynamically (Figure 1(c)). Without the help of acceptors, the bipolar character was achieved by facilitating resonance enantiomer transitions in the D-r-D architecture using the N-P = X (X = O, S, or Se) resonance linkers, which is significantly different from the current widely used D-A strategy. Separated by the resonance linkage of N-P = X, the electronic coupling between two donors of carbazole is insufficient; thus, the optical properties of carbazole were preserved for large $E_{g}$ and high $E_{T}$ [19]. Therefore, the blue and deep-blue PhOLEDs hosted by these dynamically bipolar D-r-D molecules exhibit high external quantum efficiencies (EQEs) up to 16.2% and 18.3%, respectively. These novel resonance-driven bipolar molecules in D-r-D structure with high device performance illustrate a new way for designing bipolar organic optoelectronic semiconductors with high solubility, $E_{g}$, and $E_{T}$ simultaneously, which should be highly instructive *via* dynamic strategies instead of conventional static approaches.

## 2. Results

### 2.1. Molecular Design, Preparation, and Characterization

To take advantages of charge redistribution during the resonance variation, we constructed a type of new-concept bipolar molecules using carbazole as the donor and N-P = X (X = O, S, or Se) as the resonance linkage in a D-r-D configuration with an electronically inert but highly solvent soluble *tert*-butyl group on phosphine (Figure 1(d)). Owing to the inert nature of *tert*-butyl, the electron-withdrawing feature of P = X cannot tune it to be an acceptor as in the case of phenyl substituent [20]. These acceptor-free bipolar D-r-D molecules of *t*-BuPO, *t*-BuPS, and *t*-BuPSe were facilely synthesized *via* the direct N-P coupling followed by oxidation, sulfuration, or selenylation reaction in good total yields (45-72%, Scheme S1) [21]. Structure characterizations were established on the basis of ^1^H NMR, ^13^C NMR, HRMS (Figures S1-S8), elemental analysis, and single crystal analysis (Table S1). Small fractional free volumes ($V_{f}$) were observed, especially in *t*-BuPS, suggesting their compact molecular packing for strong intermolecular interactions in solid state (Figure S9) [22]. Good thermal stability and excellent film-forming property of these D-r-D molecules were revealed by thermogravimetry analyses (TGA)/differential scanning calorimetry (DSC) (Figure S10) and atomic force microscopy (AFM) (Figure S11), respectively; the decomposition temperatures ($T_{d}\text{s}$) up to 296°C and root-mean-square roughness (RMS) lower than 0.281 nm are favorable in the fabrication of thermally and morphologically stable thin films for optoelectronic devices [23, 24].

### 2.2. Resonance Variability Engineering

**Figure 2:**
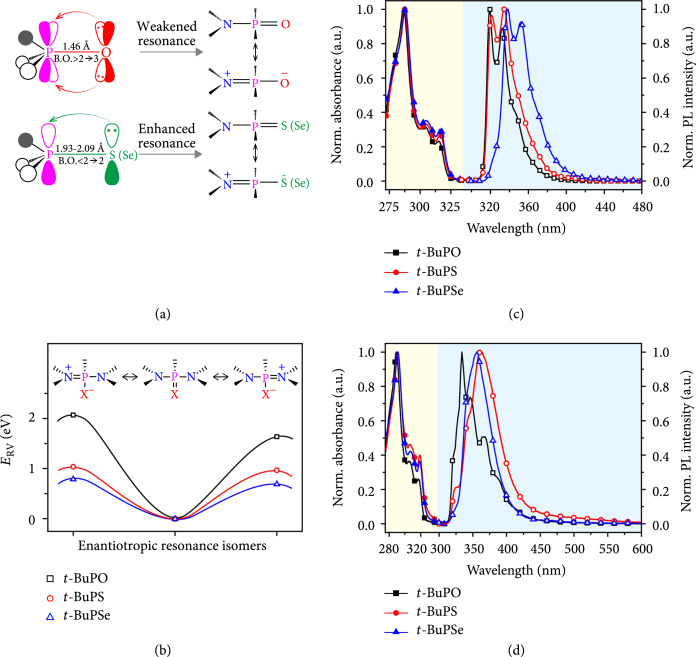
Resonance variability and photophysical properties of the D-r-D molecules. (a) Correlation between bond and resonance variations of N-P = X (X = O, S, or Se). B.O. refers to bond order. (b) Theoretical activation energy of resonance variation ($E_{\text{RV}}$) between N-P = X and N^+^ = P-X^-^ resonance isomers. (c, d) UV-absorption (solid symbols) and photoluminescence (PL) spectra (open symbols) of *t*-BuPO, *t*-BuPS, and *t*-BuPSe in (c) CH_2_Cl_2_ solution (~10^-5^ mol L^-1^) and (d) solid films.

Single-crystal X-ray diffraction analysis (Figure 1(d) and Table S1 and S2) of these newly prepared D-r-D molecules shows that the two N-P bond lengths around 1.71 and 1.69 Å are asymmetric and remarkably shorter than the normal bond length of N-P (1.76 Å) but longer than that of N=P (1.58 Å) [25, 26]. These experimental geometrical data verify that the N-P bonds in the resonance linkage behave partially characteristics of N=P, reflecting that N^+^ = P-X^-^ resonance isomers are the significant components of the molecular configuration (Scheme S2). The shortest N-P bonds of *t*-BuPSe suggest its largest tendency in forming enantiotropy N^+^ = P-Se^-^ resonance structures. These understandings can be further confirmed theoretically by the asymmetric N-P bond lengths and bond order (B.O.) analysis through the density functional theory (DFT) calculations (Figure 2(a) and Tables S3 and S4) [18, 27]. The bond orders of N-P were figured out to be around 1.18 in the D-r-D molecules, while that of P = X are 2.12 for *t*-BuPO, 1.83 for *t*-BuPS, and 1.74 for *t*-BuPSe, demonstrating quantitatively the coexistence and relative contents of the N-P = X and N^+^ = P-X^-^ canonical forms. This increased resonance variation from N-P=O to N-P=Se with the weakened P = X bonds and the decreasing bond orders is in line with that found from the localized-orbital locator (LOL) profiles (Figure S11), which show the lowest bond delocalization of P=Se for its strongest resonance variation in the D-r-D molecules [28, 29]. Moreover, natural bond orbital (NBO) analysis [30] reveals that the injected charges, either hole or electron, are mainly delocalized on the two carbazole units with a minor dispersion on the *tert*-butyl P = X moiety (Table S3), indicating clearly that under the manipulation of the resonance variation with significant charge redistribution, the carbazole is tuned to be capable of both hole and electron injection and transport. These results are rational that the considerable capacity on opposite charges for carbazole is supported by the polarized canonical form of N^+^ = P-X^-^ during resonance variation to significantly modify the electronic feature of carbazole, which can be hardly realized in conventional D-A systems.

To accurately estimate of resonance variation ability of the D-r-D molecules for the dynamic bipolar characteristics, the energy difference of the idealized natural Lewis structures of N-P = X and N^+^ = P-X^-^, defined as the activation energy of resonance variation ($E_{\text{RV}}$), was calculated by deleting all Fock matrix elements between Lewis NBOs and the vicinal non-Lewis NBOs (Figure 2(b) and Table S5) [18, 31]. The $E_{\text{RV}}\text{s}$ of *t*-BuPO (2.05 and 1.64 eV), *t*-BuPS (0.97 and 1.04 eV), and *t*-BuPSe (0.67 and 0.79 eV) show the decreased energy barriers for the enhanced resonance variation between the N-P = X and N^+^ = P-X^-^ canonical forms, indicating again the facilely engineered resonance variability of the D-r-D molecules by simply changing X from O to Se atoms. The more superior resonance variability of *t*-BuPS and *t*-BuPSe than that of *t*-BuPO and their almost negligible energy difference between the two enantiotropic N^+^ = P-X^–^ resonances (~0.07 eV) suggest the nearly barrier-free resonance variation of these two canonical atoms; thus, the two carbazoles could have very rapid and self-adaptive switch in hole or electron transporting features for more balanced charge injection and transport upon dynamic resonance variations.

### 2.3. Optical Properties

Photophysical properties of the D-r-D molecules were investigated by UV-vis absorption and fluorescence spectra (Figures 2(c) and 2(d)). The insulating feature of P = X (X = O, S, or Se) in these molecules results in a weak electronic communication between the conjugated carbazole units [32, 33] and almost identical absorption spectra of the three different D-r-D molecules in both dilute solutions and thin films. Nevertheless, owing to the electron-withdrawing feature of O, S, and Se atoms, the absorption peaks are slightly blue shifted from 292, 320, and 333 nm of carbazole to ~287, 304, and 315 nm of these D-r-D molecules in dichloromethane (CH_2_Cl_2_) solution. This hypochromic shift was also observed in the emission spectra, exhibiting the smallest blue shift in *t*-BuPSe (338 and 353 nm) compared to carbazole (341 and 354 nm) due to the weakest electronegativity of Se (Table S6) [17]. In solid films, the emission bands were bathochromic-shifted, and *t*-BuPS shows the largest red shift compared to that in solution, suggesting its strongest intermolecular interactions in solid film which is in line with its low free volume region with small $V_{f}$ of 4.3% (Figure S9) and heavy attractive and repulsive interactions from reduced density gradient calculations (Figure S13) [34]. The low-temperature (77 K) time-resolved phosphorescence spectra suggest that the lowest triplet excited states ($T_{1}$) energy levels of these molecules are all as high as 2.97 eV (Figure S14 and S15(a)), which are very close to that of carbazole (3.05 eV) [19]. DFT calculations confirm the dominating role of the carbazole unit in determining $T_{1}$ from the well-controlled spin density distribution on the carbazole (Figure S15(b)). It should be also noted that the D-r-D molecules show no obvious intramolecular charge transfer (ICT) feature from the emission spectra in different solvents with varied polarities (Figure S16), because of the absence of acceptor units. Therefore, the carbazole chromophore dominates all the optical features, resulting in very similar photophysical properties of these D-r-D molecules.

### 2.4. Electronic Properties

**Figure 3:**
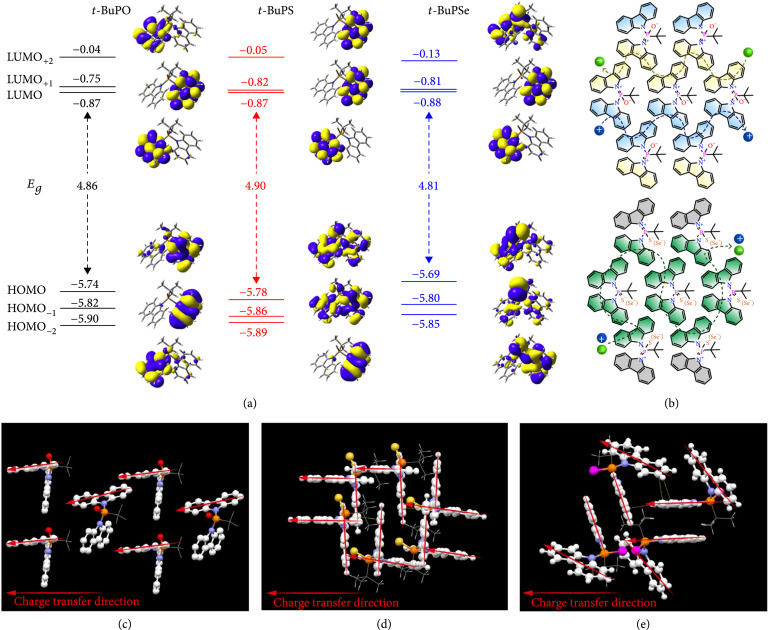
Electronic properties of the D-r-D molecules. (a) Frontier molecular orbital energy levels and distributions, (b) charge injection and transport models, and potential carrier transport styles of (c) *t*-BuPO, (d) *t*-BuPS, and (e) *t*-BuPSe on the basis of their single-crystal packing modes.

Electrochemical properties of the D-r-D molecules were studied by cyclic voltammetry (CV) experiments (Figure S17). From the onset of the oxidative wave, the highest occupied molecular orbital (HOMO) energy levels were identified to be very close (−6.15±0.01 eV), and with the aid of the optical $E_{g}\text{s}$, the lowest unoccupied molecular orbital (LUMO) of these molecules was deduced to be also very similar (−2.31±0.01 eV) [35]. DFT calculations are well in line with the experimental results, revealing that the carbazole unit dominates both the HOMOs and LUMOs of the D-r-D molecules; this should be the exact reason for their very close frontier orbital energy levels (Figure 3(a)). More importantly, this gives an important evidence for the formation of D-r-D molecular structure by simply changing phenyl to *tert*-butyl on P = X; the small contributions of P = X to LUMO in *t*-BuPX suggest that P = X can only behave as an electron-withdrawing unit here, although it is generally considered as an acceptor in many studies [36, 37]. The dominated role of carbazole unit in controlling both electron and hole injection and transport was further confirmed by the NBO analysis of the positively and negatively charged molecules (Table S3). The injected charges are mainly located on carbazoles with very small distributions on *tert*-butyl and P = X. The different resonance variabilities of N-P=O, N-P=S, and N-P=Se linkages would result in different carrier transport behaviors of the D-r-D molecules (Figure 3(b)). For *t*-BuPO, the resonance variation between its neutral and two enantiotropic polarized forms is relatively difficult compared to other two resonance molecules, leading to retardative self-adaptively in balancing carrier transport upon the environmental stimuli; this would restrain the host-to-dopant charge transfer and carrier recombination in device applications. Specifically, in the packing diagram of *t*-BuPO crystal (Figure 3(c)), the distance of two adjacent molecules is quite large; the carriers could be difficult to transport in the solid state of this molecule, rendering the deteriorated effects on device operation [38]. In contrast, the facile resonance variation and almost equivalent enantiotropic isomers of N^+^ = P-S^–^ and N^+^ = P-Se^–^-based resonances can support the rapid switching between the neutral and polarized resonance forms to dynamically generate the long-range and large-scale channels for both efficient hole and electron transport [18]. The single crystal diagrams of *t*-BuPS and *t*-BuPSe with compact and interlaced molecular packing style further support the strong intermolecular interaction in solid state (Figure 2(d)) and the facile carrier transporting network to enhance the carrier flux balance and charge carrier recombination for improving electroluminescent (EL) performance (Figures 3(d) and 3(e)).

### 2.5. Bipolar Carrier Transport Properties

**Figure 4:**
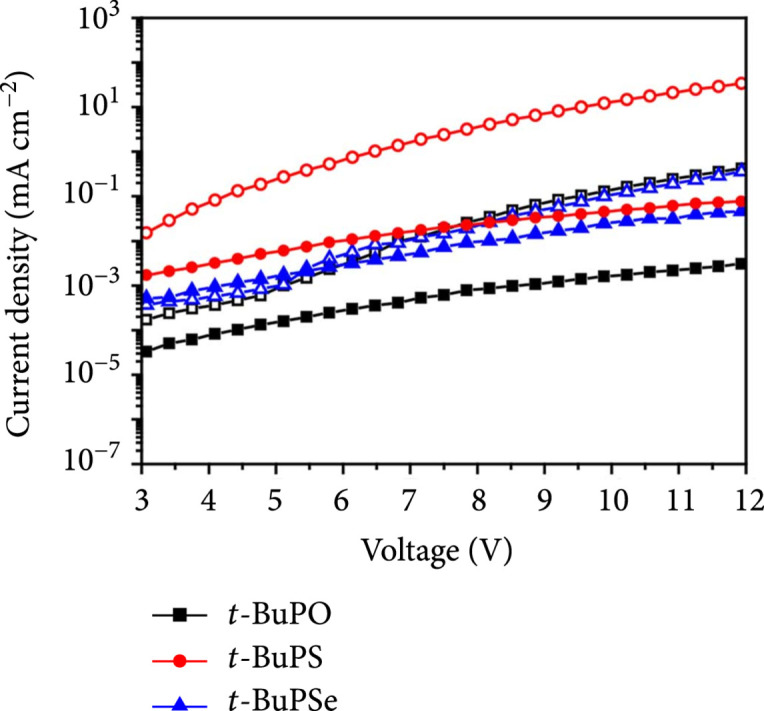
Bipolar carrier transport properties of the D-r-D molecules. Current-voltage (J-V) characteristics of the single-carrier transporting devices based on the D-r-D molecules (solid and open symbols are for the electron-only and hole-only devices, respectively).

To experimentally evaluate the effects of the multidimensional dynamic channels for carrier transport by tuning the whole molecule capable of both hole and electron transport through resonance linkage, normal single-carrier transporting devices of the D-r-D molecules were investigated (Figure 4) [35]. All these molecules exhibit hole-dominant characteristics with larger hole-only current densities (J) than the electron-only J. The lowest electron-only J was found in *t*-BuPO, which has the lowest resonance variability to dynamically tune the hole-transporting carbazole to transport electron *via* N^+^ = P-O^–^ resonance isomers. Meanwhile, *t*-BuPSe, which has the highest resonance variability with facile charge transport networks, shows the most balanced carrier transporting ability with nearly equivalent current densities in its hole-only and electron-only devices, suggesting the close relation between the hole and electron transport balance and the $E_{\text{RV}}$ of D-r-D molecules. Nevertheless, the highest hole-only and electron-only J were observed in *t*-BuPS, probably due to the combined effects of its small $E_{\text{RV}}$ for high resonance variation, delocalized HOMO distribution on both carbazoles for significantly increased hole-only J, and low reorganization energies for both facile hole and electron transport (Table S7) and the lowest $V_{f}$ for strong intermolecular interaction (Figure S9). The hole and electron transporting mobilities of the D-r-D molecules are up to 9.18∗10^-6^ and 1.12∗10^-7^ cm^2^V^-1^ s^-1^, respectively. It should be noted that these values are comparable to the mobilities of traditional D-A type bipolar materials (Table S7) [14], suggesting unambiguously the intrinsic effects of resonance variation on achieving enhanced and balanced bipolar charge transport of organic semiconductors even without the participation of electron acceptor units.

### 2.6. Device Applications in PhOLEDs

**Figure 5:**
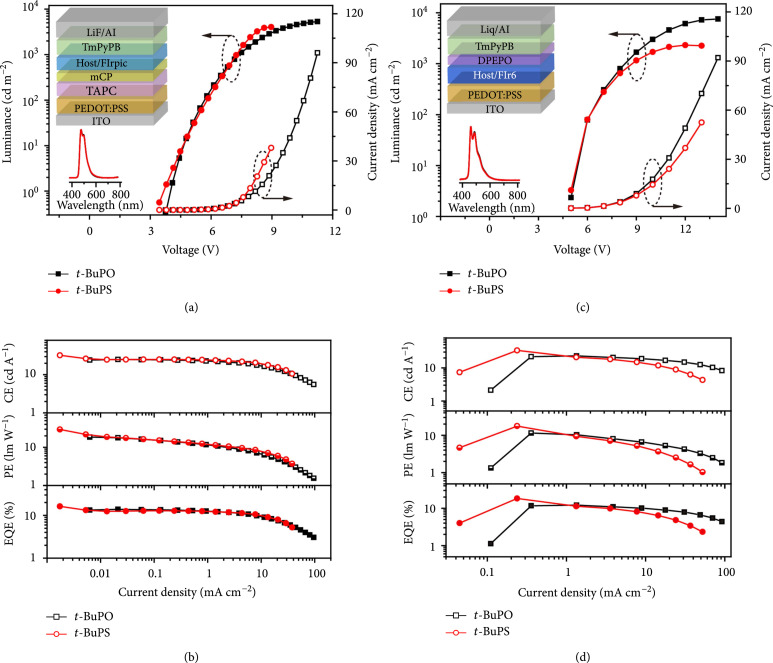
EL performance of the blue and deep-blue PhOLEDs hosted by the D-r-D molecules. (a, c) Current density-voltage (open symbols) and luminance-voltage (solid symbols) curves and (b, d) efficiency-current density curves of the vacuum-deposited FIrpic-based blue (a, b) and solution-processed FIr6-based deep-blue (c, d) PhOLEDs hosted by *t*-BuPO and *t*-BuPS. Insets: device configuration (upper) and EL spectra at 7.0 V (lower).

In light of the excellent bipolar charge transporting property of the D-r-D molecules with high $E_{T}$, blue PhOLEDs based on the widely used sky-blue phosphor of (bis(2-(4,6-difluorophenyl)pyridyl-N,C2′) iridium(III) picolinate (FIrpic) were fabricated by vacuum deposition in a common configuration using *t*-BuPO and *t*-BuPS as host materials for Devices A and B, respectively (Figure S18) [18, 39]. Due to the heavy quenching effects of Se, *t*-BuPSe is not suitable as materials of OLEDs, although it has the most balanced charge transport property [23]. Stable and pure emission with Commission Internationale de l’Eclairage (CIE) coordinates of (0.14, 0.31) and (0.15, 0.34) from FIrpic was observed in the EL spectra of the *t*-BuPO and *t*-BuPS-hosted devices (Figure 5(a)), indicating the complete energy transfer from the host to the dopant and efficient confinement of excitons in the emitting layers. Notably, with the feasibility of the N-P = X resonance for dynamic adaptation of electronic processes, *t*-BuPO- and *t*-BuPS-hosted blue PhOLEDs exhibit the excellent device performances with the maximum current efficiencies (CEs) of 25.2 and 32.4 cd A^-1^, power efficiencies (PEs) of 18.6 and 29.5 lm W^-1^, and EQEs of 13.9 and 16.2%, respectively (Figure 5(b) and Table 1). These efficiencies of the D-r-D host materials are readily close to the conventional D-r-A hosts (EQE=16.7%) in the same device structure [18], demonstrating an even more spectacular contributions of the resonance variation to dynamically improve and balance charge transporting without the help of any acceptor units.

**Table 1:** Device performances of the vacuum-deposited blue (A and B) and solution-processed deep-blue (C and D) PhOLEDs hosted by the D-r-D molecules.

Device	Host	Guest	$V_{\text{on}}$ (V)	Efficiency^a^			Efficiency roll-off (%)^b^			CIE (x, y)^c^
				CE (cd A^-1^)	PE (lm W^-1^)	EQE (%)	CE	PE	EQE	
A	*t*-BuPO	FIrpic	4.0	25.2, 23.7, 19.8	18.6, 13.2, 8.4	13.9, 13.0, 10.8	5.9, 21.4	29.0, 54.8	6.5, 22.3	(0.14, 0.31)
B	*t*-BuPS	FIrpic	3.7	32.4, 24.9, 22.1	29.5, 13.5, 9.6	16.2, 12.7, 11.3	23.1, 31.8	54.2, 67.5	21.6, 30.2	(0.15, 0.34)
C	*t*-BuPO	FIr6	4.7	22.9, 22.1, 20.2	11.5, 11.3, 7.7	12.1, 11.8, 10.7	3.5, 11.8	1.7, 33.0	2.5, 11.6	(0.17, 0.27)
D	*t*-BuPS	FIr6	4.6	33.7, 31.2, 15.8	17.6, 16.0, 5.7	18.3, 17.0, 8.6	7.4, 53.1	9.1, 67.6	7.1, 53.0	(0.17, 0.26)

^a^In the order of maximum, 100 and 1000 cd m^-2^. ^b^Roll-offs (%) of CE, PE, and EQE in the order of 100 and 1000 cd m^-2^. ^c^At 7 V.

Further, considering the excellent solubility and high $E_{T}$ up to 2.97 eV of the D-r-D molecules with the flexible and inert *tert*-butyl substituent on the resonance linkage, the solution-processed deep-blue PhOLEDs based on [bis(2,4-difluorophenylpyridinato)tetrakis(1-pyrazol-yl)borate] iridium(III) (FIr6) were constructed in Devices C and D using *t*-BuPO and *t*-BuPS as host materials, respectively (Figure S19) [40]. The $E_{T}\text{s}$ of the D-r-D molecules are higher than that of FIr6 (2.73 eV), resulting characteristic FIr6 emission in the EL spectra with CIE coordinates of (0.17, 0.27) and (0.17, 0.26) for the *t*-BuPO- and *t*-BuPS-hosted PhOLEDs, respectively (Figure 5(c)). Excitingly, the solution-processed deep-blue PhOLEDs hosted by *t*-BuPS show a maximum CE of 33.7 cd A^-1^, PE of 17.6 lm W^-1^, and EQE of 18.3% with low efficiency roll-off (Figure 5(d) and Table 1), which are even higher than that of vacuum-deposited blue PhOLEDs (Devices A and B). To the best of our knowledge, these efficiencies are among the best results of the FIr6-based PhOLEDs hosted by small molecules. The relatively lower efficiencies of Device C hosted by *t*-BuPO should be due to its lower dynamic self-adaptability for enantiotropic resonance variation in the device operation, since the requirements of host materials are more complicated and challenging to support high charge transport balance and energy transfer efficiency in solution-processed PhOLEDs with the simplified device structures. Nevertheless, the *t*-BuPO–hosted deep-blue PhOLEDs still exhibit considerably high device performance; the maximum EQEs of the device hosted by *t*-BuPO and *t*-BuPS are almost two or three folds higher than the best results of the highest EQEs of the small-molecule-hosted PhOLEDs by solution processing reported so far (Table S8 and Scheme S3).

## 3. Discussion

In summary, we proposed an effective strategy for designing dynamically bipolar organic semiconductors based on a D-r-D architecture using N-P = X (X = O, S, or Se) resonance structures. By taking advantages of the facile resonance variation, the widely recognized carbazole donor unit can be tuned to be electron transportable when positively charged in enantiotropic N^+^ = P-X^-^ canonical form. Impressively, these D-r-D molecules exhibit excellent solubility, high thermal and morphological stability, large $E_{g}$ and high $E_{T}$, matched HOMO and LUMO energy levels, and efficient and balanced bipolar charge transport properties. Moreover, the vacuum-deposited blue and solution-processed deep-blue PhOLEDs hosted by these D-r-D bipolar molecules show maximum CEs of 32.4 and 33.7 cd A^-1^, PEs of 29.5 and 17.6 lm W^-1^, and EQEs up to 16.2% and 18.3%, respectively, which are among the best results of the reported blue/deep-blue PhOLEDs. Although the design of D-r-D molecules is difficult due to the limited choice of applicable building blocks, these advances in constructing bipolar organic semiconductors should offer important guidelines for the development of new-concept organic optoelectronic molecules with dynamic features, breaking through the traditional static strategies in achieving bipolar charge transport characteristics using both donor and acceptor building blocks.

## 4. Materials and Methods

The structures of *t*-BuPO, *t*-BuPS, and *t*-BuPSe reported in this article have been deposited in the Cambridge Crystallographic Data Centre under accession numbers CCDC: 1862724, 1862725, and 1862726. Additional synthesis, crystallographic, thermal, film-forming, photophysical, electronic data, and computational methods are included in the Supplementary Materials.

## Data Availability

All other data are available from the authors upon reasonable request.

## Supplementary Materials

Supplementary Materials Figure S1: 1H NMR spectrum of *t*-BuPO in DMSO-d_6_. Figure S2: 13C NMR spectrum of *t*-BuPO in CDCl_3_. Figure S3: HRMS spectrum of *t*-BuPO. Figure S4: 1H NMR spectrum of *t*-BuPS in DMSO-d_6_. Figure S5: 13C NMR spectrum of *t*-BuPS in CDCl_3_. Figure S6: HRMS spectrum of *t*-BuPS. Figure S7: 1H NMR spectrum of *t*-BuPSe in DMSO-d_6_. Figure S8: 13C NMR spectrum of *t*-BuPSe in CDCl_3_. Figure S9: free volume region and fractional free volume ($V_{f}$) in the single crystal cells of (a) *t*-BuPO, (b) *t*-BuPS, and (c) *t*-BuPSe. Figure S10: (a) TGA and (b) DSC curves of the D-r-D molecules. Figure S11: AFM height images of the spin-coated thin films of (a) *t*-BuPO, (b) *t*-BuPS, and (c) *t*-BuPSe on ITO/PEDOT: PSS surface. Figure S12: localized orbital locator (LOL) profiles of (a) *t*-BuPO, (b) *t*-BuPS, and (c) *t*-BuPSe using Multiwfn. Figure S13: reduced density gradient (RDG) versus sign (*λ*_2_)*ρ* with the view of the RDG isosurface of (a) *t*-BuPO, (b) *t*-BuPS, and (c) *t*-BuPSe dimers. Positive charges are in red, while negative charges are in blue. Figure S14: phosphorescence spectra of the D-r-D molecules at 77 K in CH_2_Cl_2_ with a delay of 5 ms after the 290 nm excitation. Figure S15: (a) Experimental and DFT calculated results of triplet energies and (b) spin density distributions of the D-r-D molecules. Figure S16: PL spectra of (a) *t*-BuPO, (b) *t*-BuPS, and (c) *t*-BuPSe in different solvents with different polarities. The concentration of the emitters is ~10_-5_ mol L_-1_, and the excitation wavelength is at 290 nm. Figure S17: cyclic voltammograms of the D-r-D molecules in thin solid films. Figure S18: device configuration and energy level diagram of the FIrpic-doped blue PhOLEDs hosted by *t*-BuPO and *t*-BuPS. Figure S19: device configuration and energy level diagram of the solution-processed FIr6-doped deep-blue PhOLEDs hosted by *t*-BuPO or *t*-BuPS. Scheme S1: synthetic route of the D-r-D molecules: (i) n-BuLi, THF, t-BuPCl_2_, -78°C; (ii) 30% H_2_O_2_, CH_2_Cl_2_, room temperature (rt); (iii) sulfur, CH_2_Cl_2_, rt; (iv) selenium, CHCl3, 55°C. Scheme S2: main resonance structures of (a) *t*-BuPO, (b) *t*-BuPS, and (c) *t*-BuPSe. Scheme S3: molecular structures of the host materials used in the FIr6-based PhOLEDs. Table S1: crystallographic data of *t*-BuPO, *t*-BuPS, and *t*-BuPSe single crystals. Table S2: the selected bond lengths of D-r-D molecules from single crystal structures. Table S3: the selected bond lengths and charge distributions of D-r-D molecules. Table S4: fuzzy bond order analysis of the D-r-D molecules. Table S5: theoretical activation energy of the resonance variation (ERV) between N-P = X and N ^+^ =P - X^-^ resonance structures (X = O, S, and Se). Table S6: photophysical and electrochemical properties of the D-r-D molecules. Table S7: B3LYP/6-31G(d) calculated reorganization energies (*λ*h for hole, *λ*e for electron) and carrier mobilities (*μ*_h_ for hole, *μ*_e_ for electron) of the D-r-D molecules. Table S8: a brief summary of the reported device performance of the FIr6-based PhOLEDs using small molecular host materials.
