# Supplementary material for: Constructing Donor-Resonance-Donor Molecules for Acceptor-Free Bipolar Organic Semiconductors
Source: Research (Wash D C). 2021 Feb 5;2021:9525802. doi: 10.34133/2021/9525802 (PMC11014465; doi:10.34133/2021/9525802)
Supplement: Supplementary Materials — Figure S1: 1H NMR spectrum of t-BuPO in DMSO-d6. Figure S2: 13C NMR spectrum of t-BuPO in CDCl3. Figure S3: HRMS spectrum of t-BuPO. Figure S4: 1H NMR spectrum of t-BuPS in DMSO-d6. Figure S5: 13C NMR spectrum of t-BuPS in CDCl3. Figure S6: HRMS spectrum of t-BuPS. Figure S7: 1H NMR spectrum of t-BuPSe in DMSO-d6. Figure S8: 13C NMR spectrum of t-BuPSe in CDCl3. Figure S9: free volume region and fractional free volume (Vf) in the single crystal cells of (a) t-BuPO, (b) t-BuPS, and (c) t-BuPSe. Figure S10: (a) TGA and (b) DSC curves of the D-r-D molecules. Figure S11: AFM height images of the spin-coated thin films of (a) t-BuPO, (b) t-BuPS, and (c) t-BuPSe on ITO/PEDOT: PSS surface. Figure S12: localized orbital locator (LOL) profiles of (a) t-BuPO, (b) t-BuPS, and (c) t-BuPSe using Multiwfn. Figure S13: reduced density gradient (RDG) versus sign (λ2)ρ with the view of the RDG isosurface of (a) t-BuPO, (b) t-BuPS, and (c) t-BuPSe dimers. Positive charges are in red, while negative charges are in blue. Figure S14: phosphorescence spectra of the D-r-D molecules at 77 K in CH2Cl2 with a delay of 5 ms after the 290 nm excitation. Figure S15: (a) Experimental and DFT calculated results of triplet energies and (b) spin density distributions of the D-r-D molecules. Figure S16: PL spectra of (a) t-BuPO, (b) t-BuPS, and (c) t-BuPSe in different solvents with different polarities. The concentration of the emitters is ~10-5 mol L-1, and the excitation wavelength is at 290 nm. Figure S17: cyclic voltammograms of the D-r-D molecules in thin solid films. Figure S18: device configuration and energy level diagram of the FIrpic-doped blue PhOLEDs hosted by t-BuPO and t-BuPS. Figure S19: device configuration and energy level diagram of the solution-processed FIr6-doped deep-blue PhOLEDs hosted by t-BuPO or t-BuPS. Scheme S1: synthetic route of the D-r-D molecules: (i) n-BuLi, THF, t-BuPCl2, -78°C; (ii) 30% H2O2, CH2Cl2, room temperature (rt); (iii) sulfur, CH2Cl2, rt; (iv) selenium, [file 9525802.f1.zip › Supplementary Materials/tBuP-Research_SI-12.0.docx]

Supporting Information

Constructing Donor-resonance-Donor Molecules for Acceptor-Free Bipolar Organic Semiconductors

He Jiang^1†^, Jibiao Jin^1†^, Zijie Wang^1^, Wuji Wang^1^, Runfeng Chen^1^, Ye Tao^1^, Qin Xue^3^, Chao Zheng^1^, Guohua Xie^2^, and Wei Huang^1,4^

Content

[1. Materials and Synthesis 2](#_Toc527283120)

[2. Single crystal X-ray analysis 9](#_Toc527283121)

[3. Thermal stabilities and film-forming properties 11](#_Toc527283122)

[4. Main resonance structures 13](#_Toc527283123)

[5. Computational methods 13](#_Toc527283124)

[6. Optical Properties 17](#_Toc527283125)

[7. Electrochemical Properties 19](#_Toc527283126)

[8. Devices fabrication and measurements 20](#_Toc527283127)

**1. Materials and Synthesis**

**Materials**: All materials, unless otherwise specified, were purchased from commercial suppliers and used without further purification. Manipulations involving air-sensitive reagents were performed in an atmosphere of dry argon during the preparation of the donor-resonance-donor (D-r-D) molecules. Tetrahydrofuran (THF) was dried and purified by routine procedures. Organic solvents, such as toluene, dichloromethane (CH_2_Cl_2_), chloroform (CHCl_3_), and dimethylformamide (DMF), were used as received without further purification.

**Instruments**: ^1^H and ^13^C-nuclear magnetic resonance (NMR) spectra were recorded on a Bruker Ultra Shield Plus 400 MHz instrument with DMSO-*d*_6_ or CDCl_3_ as the solvents and tetramethylsilane (TMS) as the internal standard. The quoted chemical shifts are in ppm and the *J* values are expressed in Hz. The splitting patterns have been designed as follows: s (singlet), d (doublet), t (triplet), dd (doublet of doublets), and m (multiplet). High resolution mass spectra were recorded on a LCT Premier XE (Waters) HRMS spectrometry. Melting points (m.p.) were determined using a SGW X-4 micro-melting point apparatus.


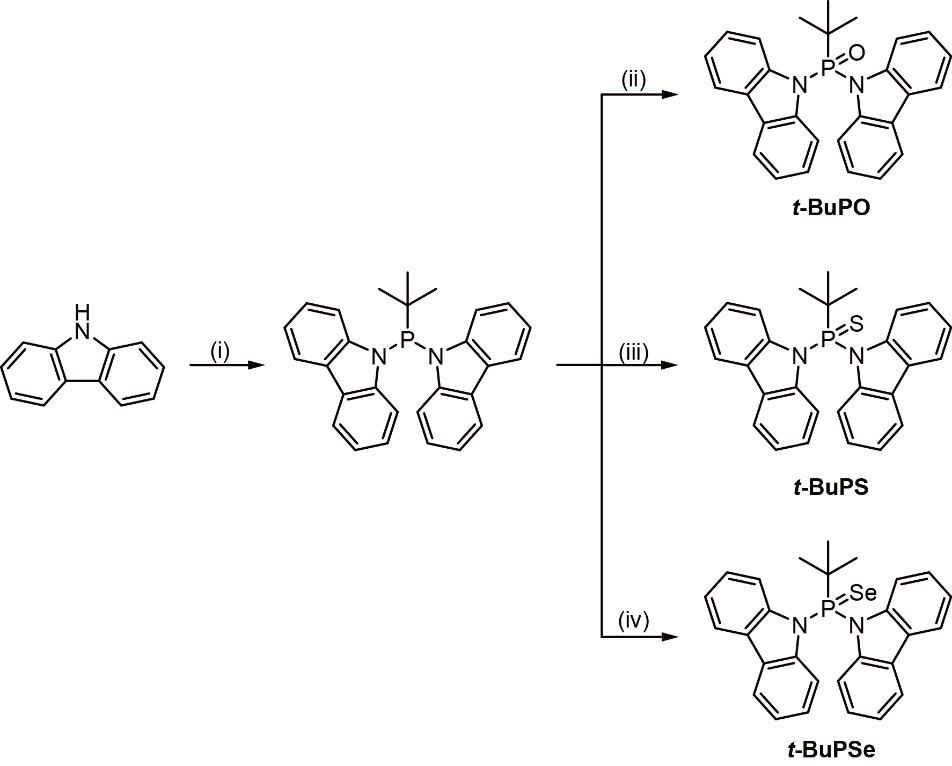


**Scheme S1.** Synthetic route of the D-r-D molecules: (i) *n*-BuLi, THF, *t*-BuPCl_2_, -78°C; (ii) 30% H_2_O_2_, CH_2_Cl_2_, room temperature (rt); (iii) sulfur, CH_2_Cl_2_, rt; (iv) selenium, CHCl_3_, 55°C.

**Synthesis of di (9*H*-carbazol-9-yl) *tert*-butylphosphine oxide (*t*-BuPO):**

To a freshly distilled THF (30 mL) solution of carbazole (1.5 g, 8.97 mmol) at -78°C under nitrogen atmosphere was added dropwise a hexane solution of *n*-butyl lithium (4.3 mL, 10.8 mmol, 2.5 M in hexane). After the lithiation at -78°C for 1 h, *tert*-butyl dichlorophosphine (*t*-BuPCl_2_) (0.1 mL, 4.5 mmol) was added into the reaction system rapidly. The reaction mixture at -78°C was allowed to warm to room temperature and stirred overnight. Then, the reaction was quenched with water (10 mL) and extracted with dichloromethane (CH_2_Cl_2_) (3×30 mL). The organic layers were collected and dried with anhydrous Na_2_SO_4_. The organic solvent was removed under reduced pressure. The residue without further purification was dissolved in dichloromethane (30 mL) and 30% hydrogen peroxide (H_2_O_2_) (0.3 g, 8.97 mmol) was added into for the following oxidation.^[1]^ After the reaction mixture was stirred overnight at room temperature, water (10 mL) was added to quench the reaction. The mixture was extracted with CH_2_Cl_2_ (3×30 mL) and the collected organic layers were dried with anhydrous Na_2_SO_4_. The organic solvent was removed under reduced pressure. The resulting crude product was purified by flash column chromatography on silica gel. Yield: 72%, white powder. m.p.: 276.8°C. ^1^H NMR (DMSO- *d*_6_, 400 MHz) *δ* (ppm): 8.18-8.16 (m, 4H), 7.60-7.57 (m, 4H), 7.29-7.26 (m, 8H), 1.60 (d, *J*=20 Hz, 9H). ^13^C NMR (CDCl_3_, 100 MHz) *δ* (ppm): 141.11, 141.08, 126.61, 126.48, 126.42 122.32, 119.90, 114.97, 39.89, 38.85, 26.43. HRMS (EI): m/z calcd for C_28_H_25_N_2_PO [M+Na]^+^: 459.1602; found: 459.1601. Anal. calcd for C_28_H_25_N_2_PO: C 77.05, H 5.77, N 6.42; found: C found: C 77.07, H 5.68, N 6.16.


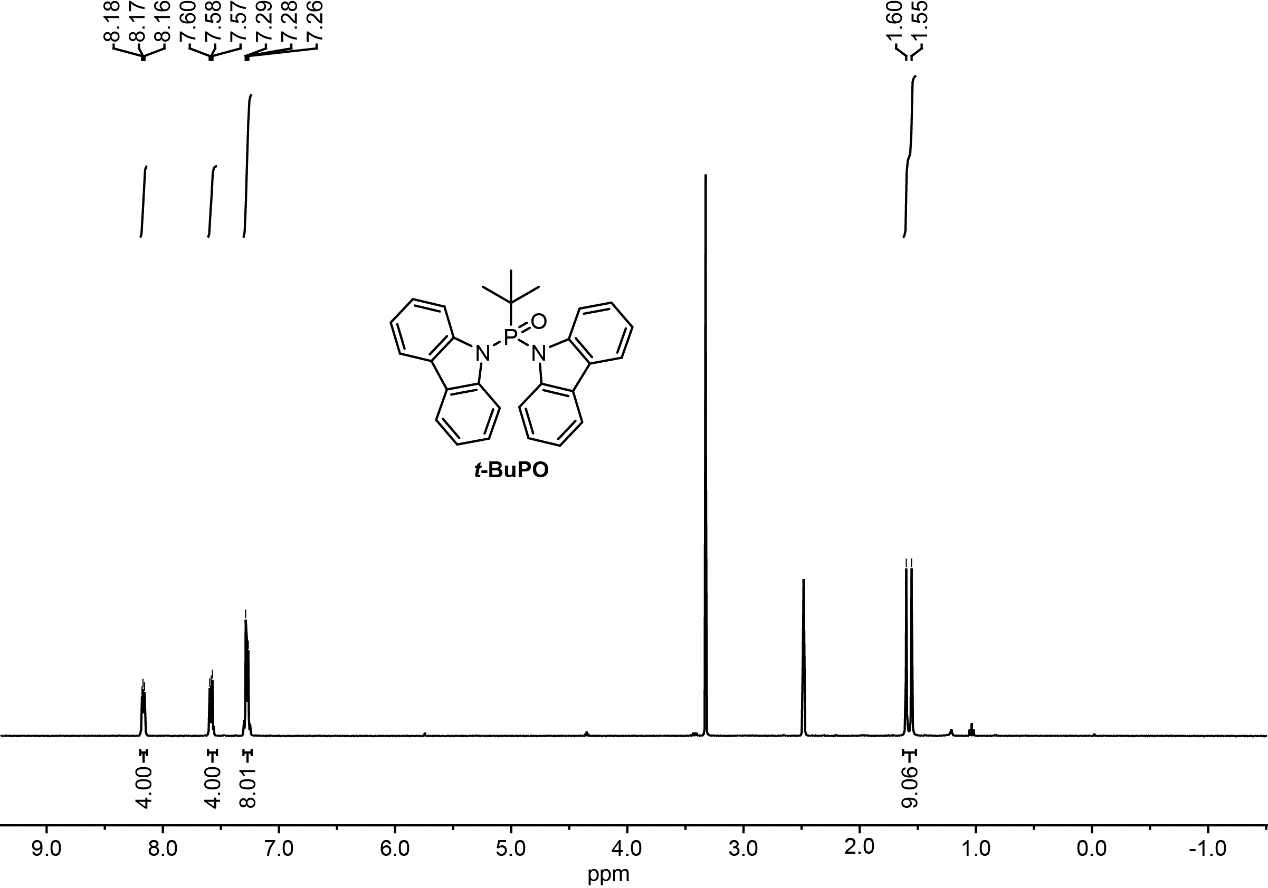


**Figure S1.** ^1^H NMR spectrum of ***t*-BuPO** in DMSO- *d*_6_.


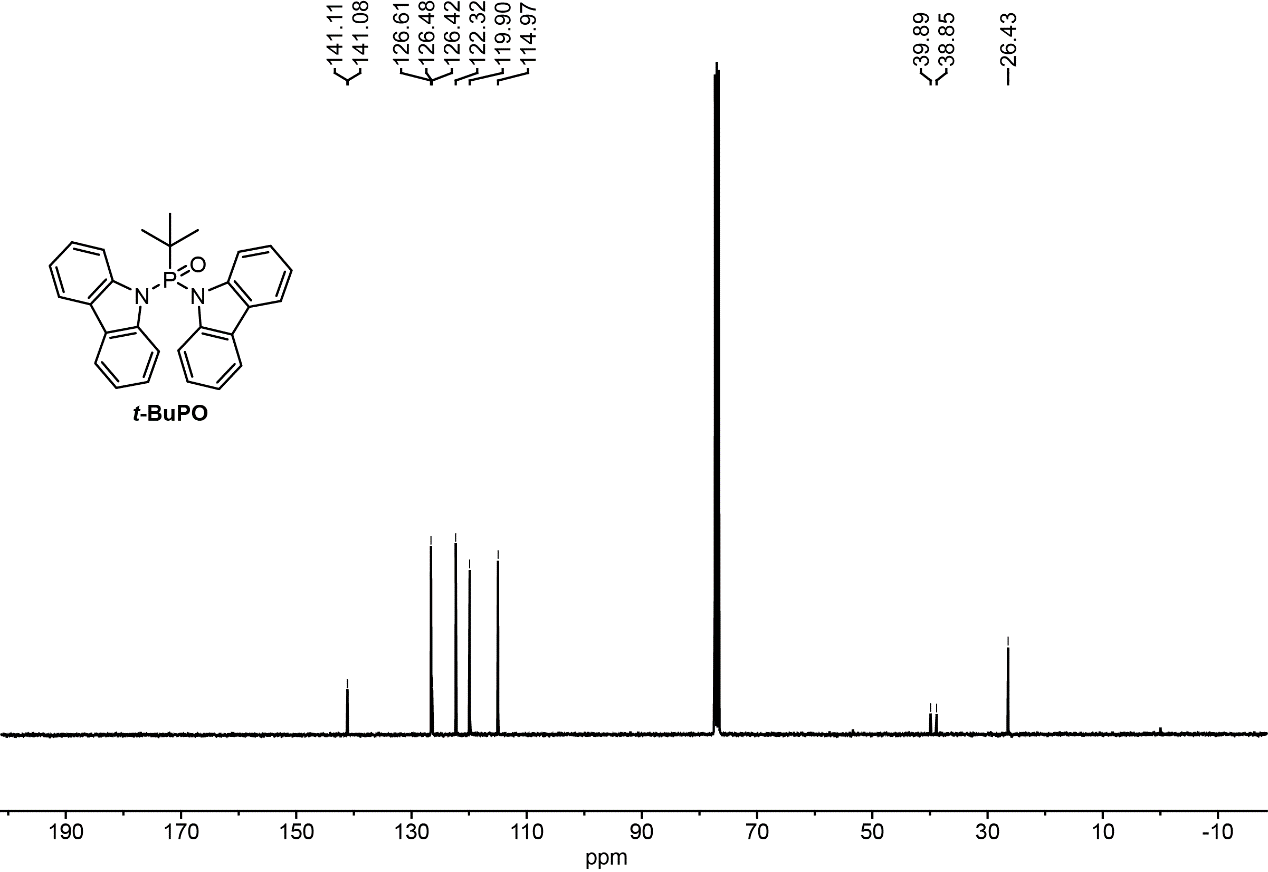


**Figure S2.** ^13^C NMR spectrum of ***t*-BuPO** in CDCl_3_.


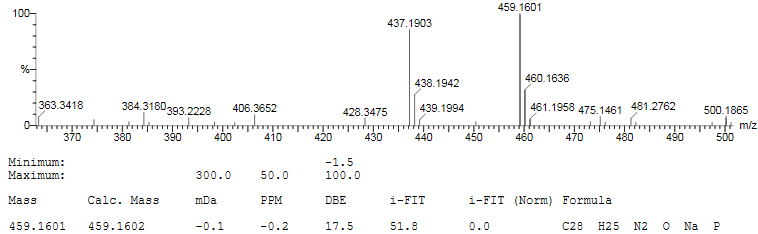


**Figure S3.** HRMS spectrum of ***t*-BuPO**.

**Synthesis of di (9*H*-carbazol-9-yl) *tert*-butyl****phosphine sulfide (*t*-BuPS):**

***t*-BuPS** was prepared in an identical synthetic procedure in preparing ***t*-BuPO** using carbazole (1.5 g, 8.97 mmol), *n*-butyl lithium (4.3 mL, 10.8 mmol, 2.5 M in hexane), and *t*-BuPCl_2_ (0.1 mL, 4.5 mmol). The resulted residue of di (9*H*-carbazol-9-yl) *tert*-butylphosphine without purification was dissolved in CH_2_Cl_2_ (30 mL) and sulfur (0.43 g, 13.5 mmol) was added into for the following sulfurization.^[2]^ Yield: 53%, white powder. m.p.: 245.0℃. ^1^H NMR (DMSO- *d*_6_, 400 MHz) *δ* (ppm): 8.18 (d, *J*=8 Hz, 4H), 7.61 (d, *J*=8 Hz, 4H), 7.29-7.20 (m, 8H), 1.74 (d, *J*=20 Hz, 9H). ^13^C NMR (CDCl_3_, 100 MHz) *δ* (ppm): 141.10, 141.07, 126.85, 126.79, 126.22, 122.34, 119.77, 115.78, 46.06, 45.31, 28.10. HRMS (EI): m/z calcd for C_28_H_25_N_2_PS [M+H]^+^: 453.1554; found: 453.1548. Anal. calcd for C_28_H_25_N_2_PS: C 74.31, H 5.57, N 6.19; found: C 74.43 H 5.54, N 5.94.


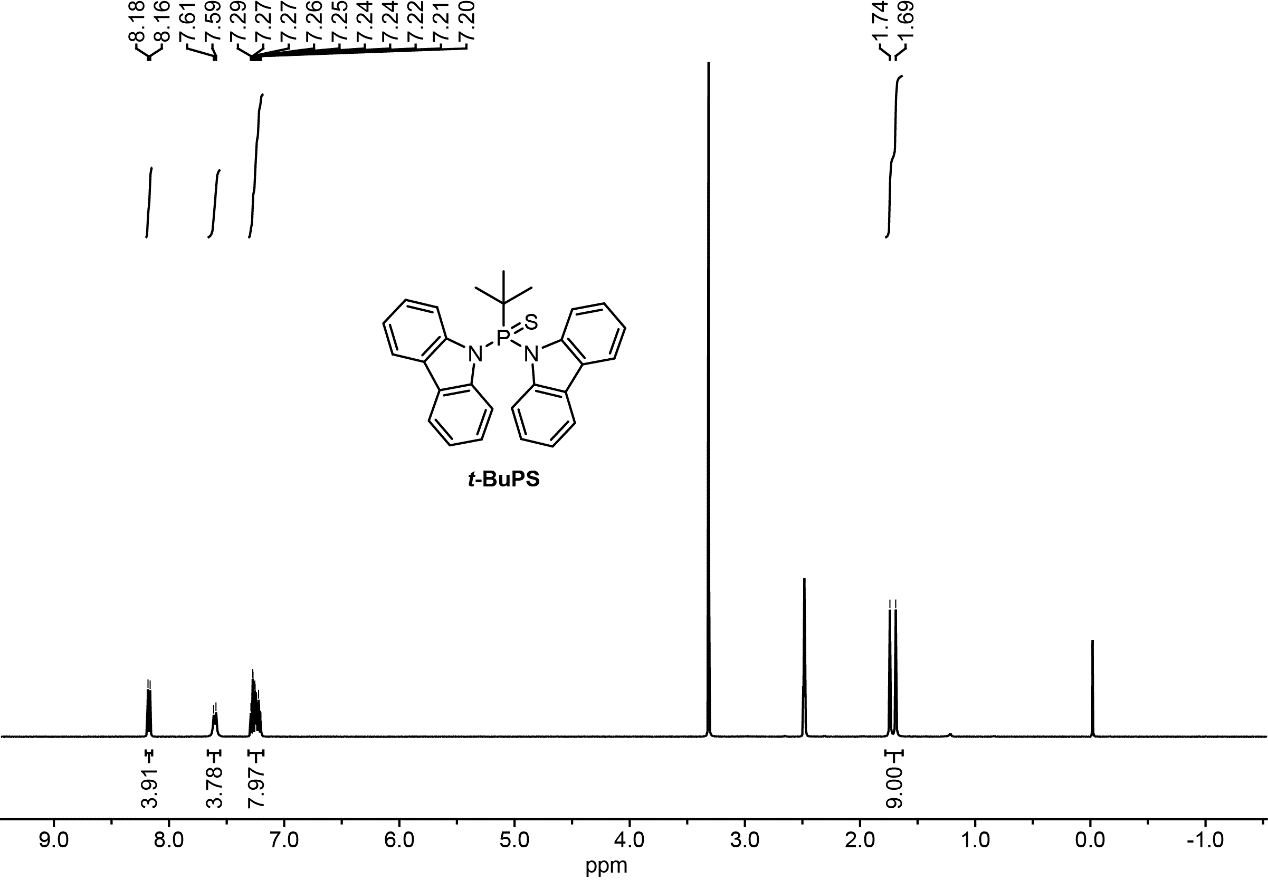


**Figure S4.** ^1^H NMR spectrum of ***t*-BuPS** in DMSO- *d*_6_.


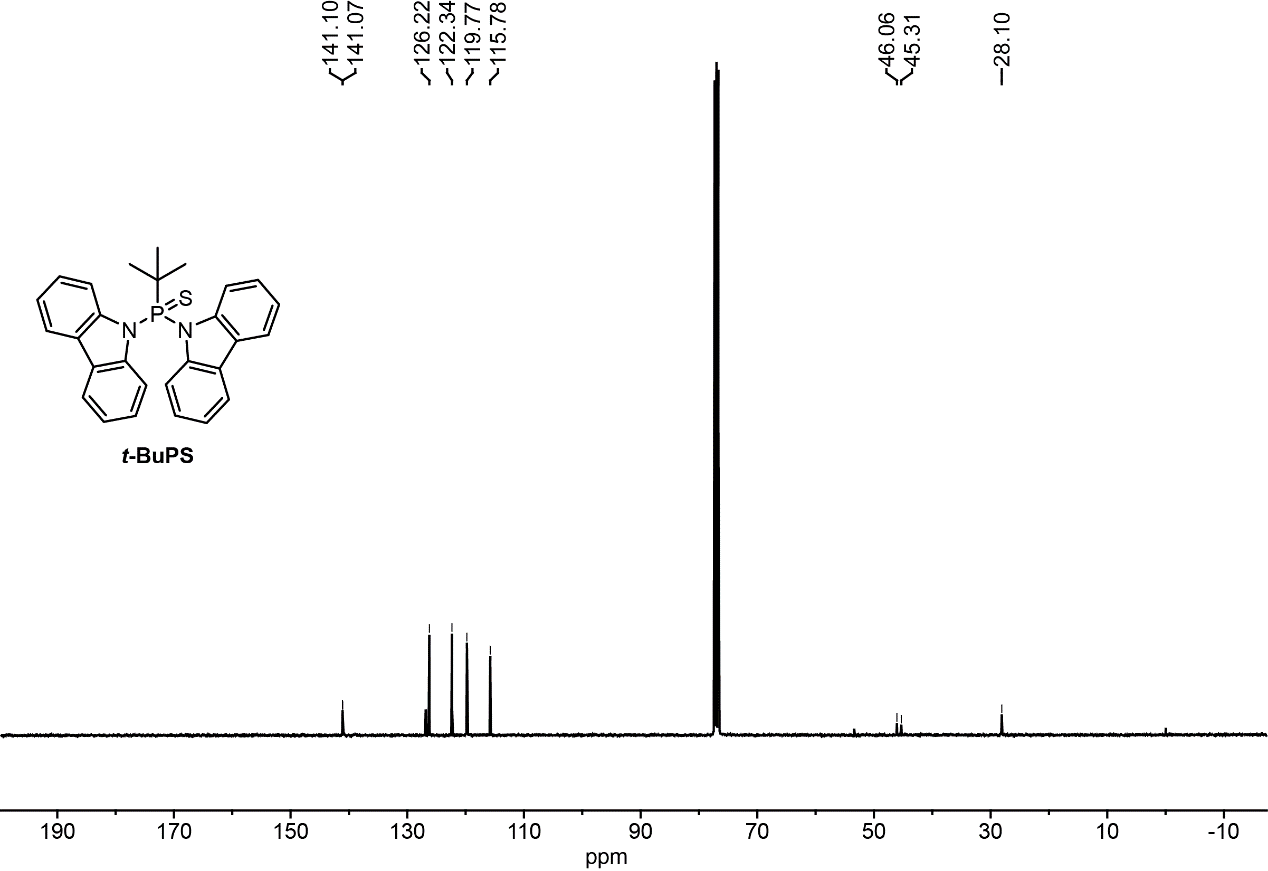


**Figure S5.** ^13^C NMR spectrum of ***t*-BuPS** in CDCl_3_.


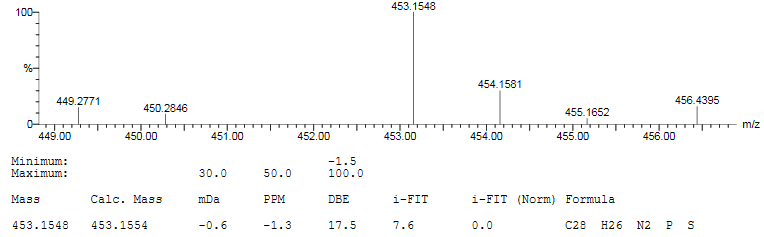


**Figure S6.** HRMS spectrum of ***t*-BuPS**.

**Synthesis of di (9*H*-carbazol-9-yl) *tert*-butylphosphine selenide (*t*-BuPSe):**

***t*-BuPSe** was prepared in an identical synthetic procedure in preparing ***t*-BuPO** using carbazole (1.5 g, 8.97 mmol), *n*-butyl lithium (4.3 mL, 10.8 mmol, 2.5 M in hexane), and *t*-BuPCl_2_ (0.1 mL, 4.5 mmol). The obtained crude di(9*H*-carbazol-9-yl) *tert*-butylphosphine was dissolved in chloroform (CHCl_3_) (30 mL) and selenium (1.05 g, 13.5 mmol) was added into for the following selenylation.^[3]^ Yield: 45%, white powder. m.p.: 230.2℃. ^1^H NMR (DMSO- *d*_6_, 400 MHz) *δ* (ppm): 8.18 (d, *J*=8 Hz, 4H), 7.63-7.61 (m, 4H), 7.29-7.20 (dt, *J*=36 Hz, 8H), 1.78 (d, *J*=20 Hz, 9H). ^13^C NMR (CDCl_3_, 100 MHz) *δ* (ppm): 141.10, 141.07, 126.85, 126.79, 126.22, 122.34, 119.77, 115.78, 46.06, 45.31, 28.10. Anal. calcd for C_28_H_25_N_2_PSe: C 67.33, H 5.05, N 5.61; found: C 67.26, H 5.34, N 5.21.


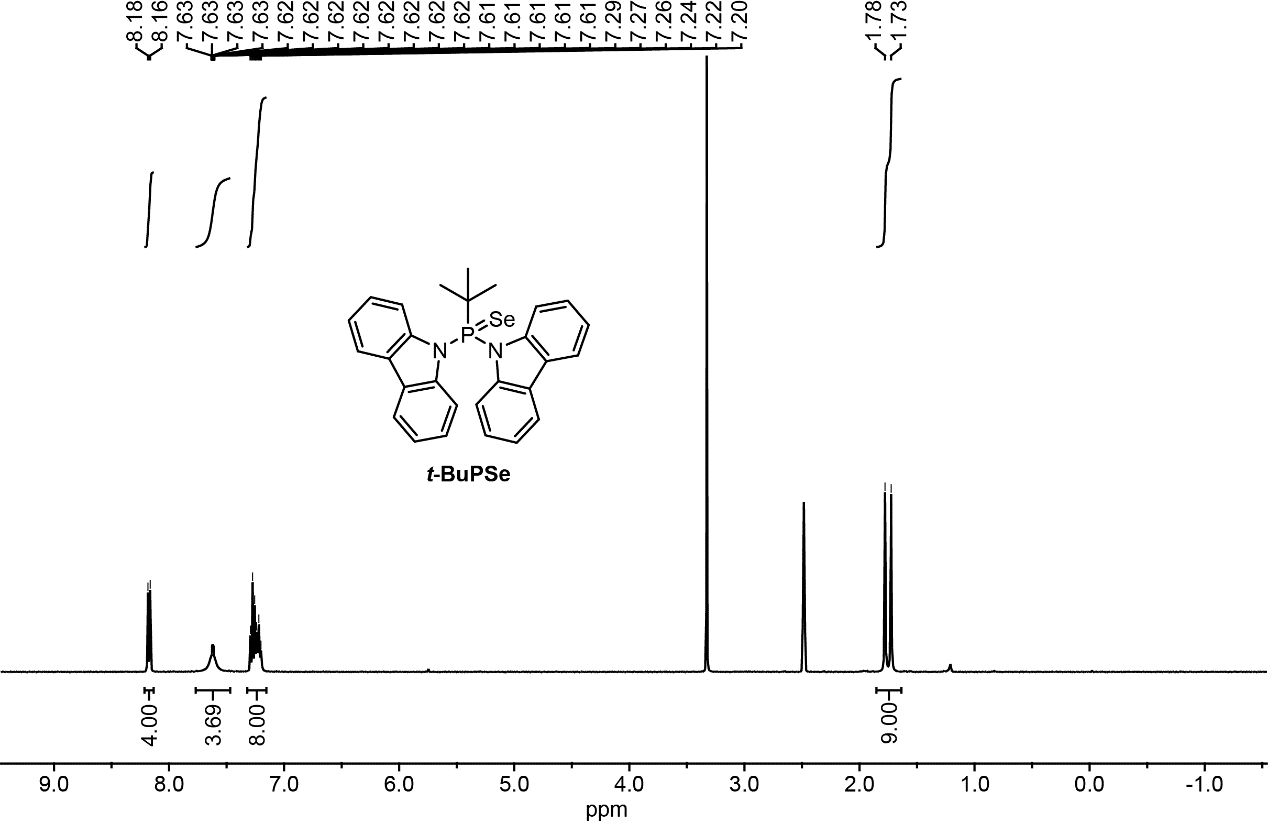


**Figure S7.** ^1^H NMR spectrum of ***t*-BuPSe** in DMSO- *d*_6_.


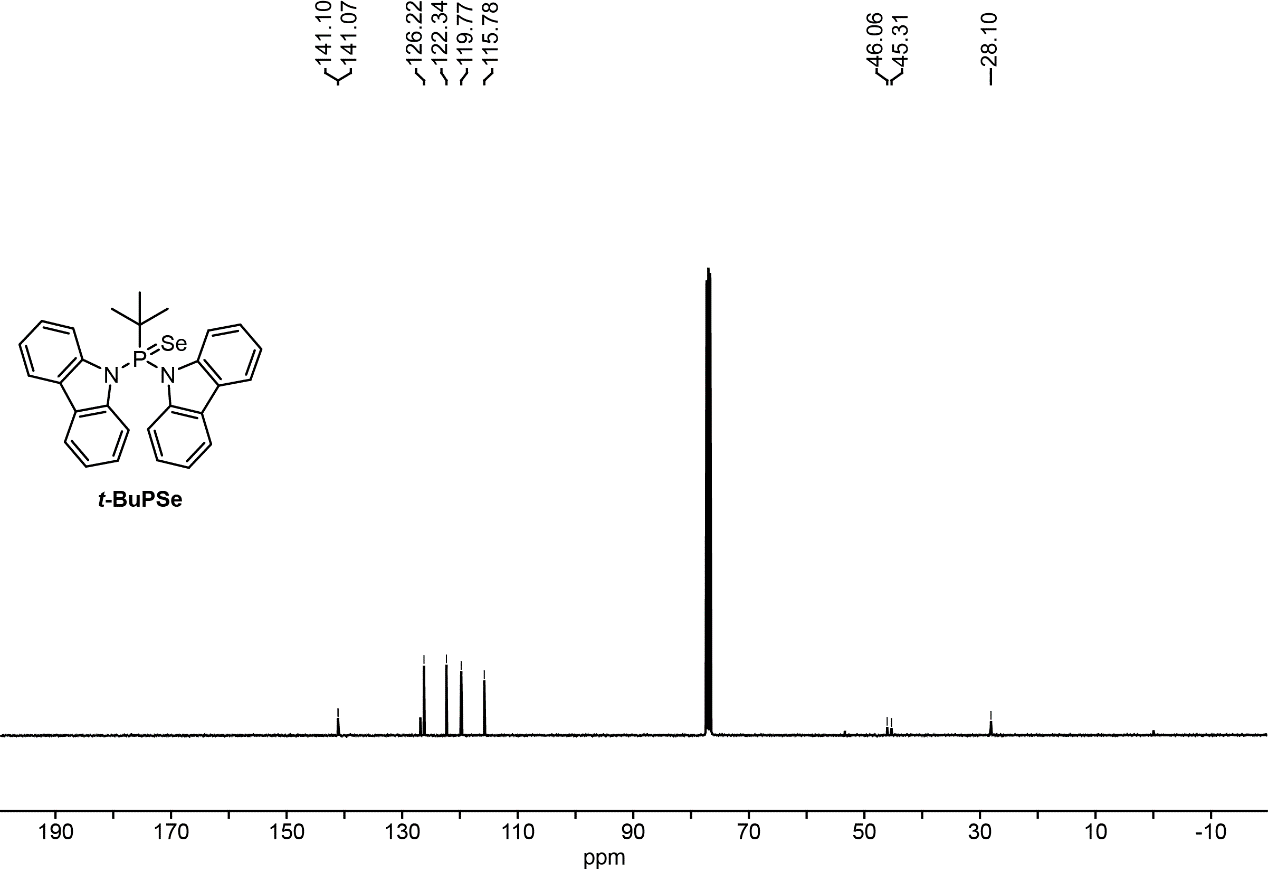


**Figure S8.** ^13^C NMR spectrum of ***t*-BuPSe** in CDCl_3_.

**2. Single crystal X-ray analysis**

Single crystals of ***t*-BuPO**, ***t*-BuPS** and ***t*-BuPSe** were grown by slow evaporation of a combined dichloromethane and ethanol solution at room temperature. Data of single crystal structures were collected on a Bruker SMART APEX (II)-CCD area detector diffractometer using graphite-monochromated Mo-Kα radiation (λ = 0.71073 Å) at 296 K. Cell parameters were retrieved using SMART software and refined using SAINT on all observed reflections. Structures were solved by direct methods with the program SHELX-97 program package. The crystal structure was analyzed by Diamond 3.2 software. Their crystallographic data were summarized in **Table S1**. The crystallographic information files (CIF) were also attached. The free volume, defined as the unoccupied volume in the single crystal cell, was calculated using Materials Studio software with a 1.0 Å Connolly radius based on the single crystal structure. Fractional free volume (*V*_f_) was the ratio of free volume to the total volume of the stimulated cell.^[5]^

**Table S1** Crystallographic data of ***t*-BuPO**, ***t*-BuPS**, and ***t*-BuPSe** single crystals.

| Compound | *t*-BuP**O** | *t*-BuP**S** | *t*-BuPS**e** |
| --- | --- | --- | --- |
| Empirical formula | C_28_H_25_N_2_PO | C_28_H_25_N_2_PS | C_28_H_25_N_2_PSe |
| Formula weight (g mol^-1^) | 436.47 | 452.53 | 499.43 |
| Crystal color | colorless | colorless | colorless |
| Wavelength (Å) | 0.71073 | 0.71073 | 0.71073 |
| Crystal system | Monoclinic | Monoclinic | Monoclinic |
| Space group | P 21/n | C1c1 | P 21/n |
| *a* (Å) | 13.997(3) | 19.275(5) | 15.381(2) |
| *b* (Å) | 9.3673(19) | 8.151(2) | 9.8830(15) |
| *c* (Å) | 34.254(7) | 16.238(4) | 15.381(2) |
| *α* (deg) | 90 | 90 | 90 |
| *β* (deg) | 92.450(5) | 119.113(5) | 99.2400 |
| *γ* (deg) | 90 | 90 | 90 |
| *V* (Å^3^) | 4487.1(15) | 2228.8(9) | 2307.7(6) |
| *Z* | 8 | 4 | 4 |
| Density (g cm^-3^) | 1.292 | 1.349 | 1.438 |
| *µ* (mm^-1^) | 0.146 | 0.237 | 1.715 |
| *T*_min_, *T*_max_ | 0.980,0.986 | 0.967,0.977 | 0.787,0.842 |
| *F*(000) | 1840.0 | 952.0 | 1024.0 |
| *h*_max_, *k*_max_, *l*_max_ | 16,11,41 | 23,10,20 | 18,11,18 |
| *Theta*_max_ | 25.499 | 25.991 | 24.996 |
| *CCDC Number* | *1862724* | *1862725* | *1862726* |

**
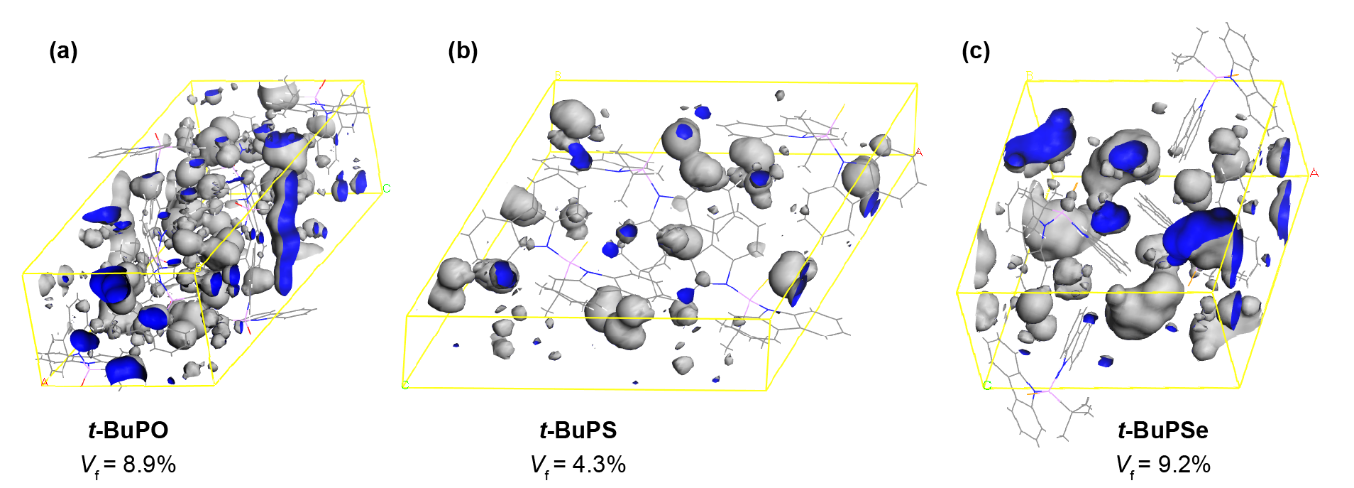
**

**Figure S9.** Free volume region and fractional free volume (*V*_f_) in the single crystal cells of (a) ***t*-BuPO**, (b) ***t*-BuPS** and (c) ***t*-BuPSe**.

**3. Thermal stabilities and film-forming properties**

Thermal properties of these compounds were investigated by thermogravimetric analysis (TGA) and differential scanning calorimetry (DSC). TGA measurements were conducted on a DTG-60 Shimadzu thermal analyst system under a heating rate of 10°C /min and a nitrogen flow rate of 50 cm^3^/min. DSC analyses were performed on a Shimadzu DSC-60A instrument under a heating rate of 10°C /min and a nitrogen flow rate of 20 cm^3^/min. Atomic force microscopy (AFM) was carried out at room temperature to test the film-forming properties using a FSM-Precision FM-Nanoview 1000 AFM equipped with a Scanasyst-Air peak force tapping mode AFM tips from Bruker. Thin films for AFM measurements were prepared in two steps: firstly, chlorobenzene solutions (10 mg/mL) of ***t*-BuPO**, ***t*-BuPS**, and ***t*-BuPSe** were spin-coated (2000 rpm) on the surface of indium tin oxides (ITO)/ poly(3,4-ethylenedioxythiophene): poly(styrenesulfonate) (PEDOT:PSS) substrates; then, these films were thermal annealed at 50℃ for 10 min. Uniform films of ***t*-BuPO**, ***t*-BuPS**, and ***t*-BuPSe** on ITO/PEDOT: PSS surface were identified by AFM images with low root mean square (RMS) roughness of 0.231, 0.206 and 0.281 nm, respectively.


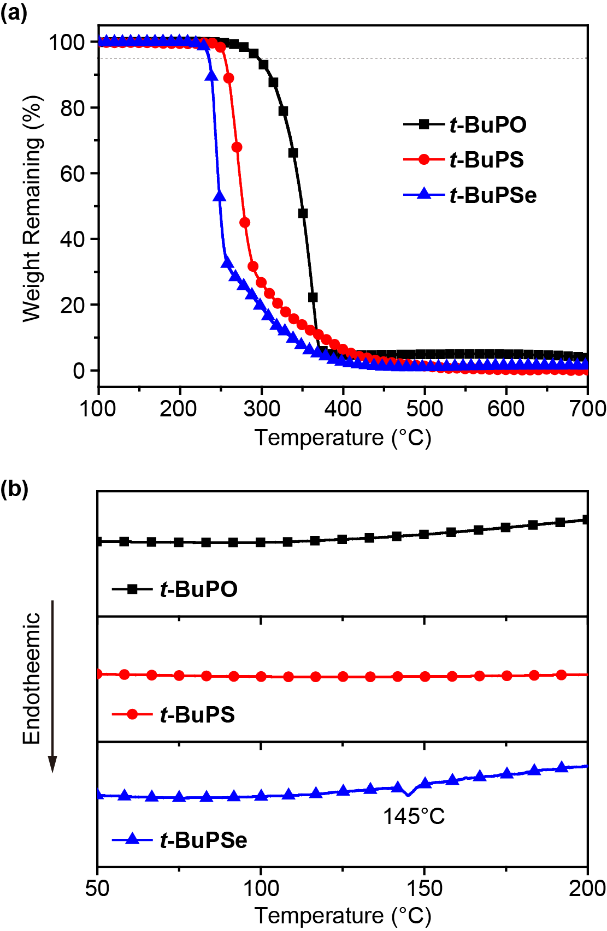


**Figure S10.** (a) TGA and (b) DSC curves of the D-r-D molecules.


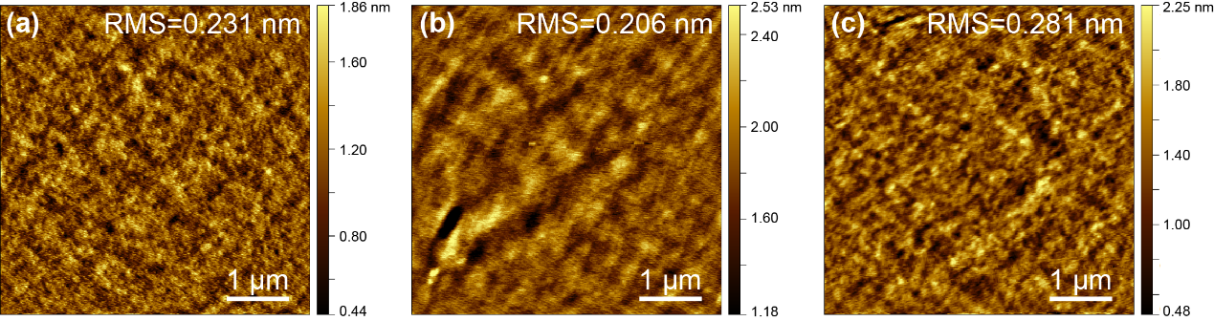


**Figure S11.** AFM height images of the spin-coated thin films of (a) ***t*-BuPO**, (b) ***t*-BuPS**, and (c) ***t*-BuPSe** on ITO/PEDOT: PSS surface.

**4. Main resonance structures**

The facile resonance interconversion of D-r-D molecules results in a large number of isomers between neutral N-P=X (X=O, S and Se) and two polarized N^+^=P-X^-^ canonical forms. The main resonance structures were illustrated in **Scheme S2**.


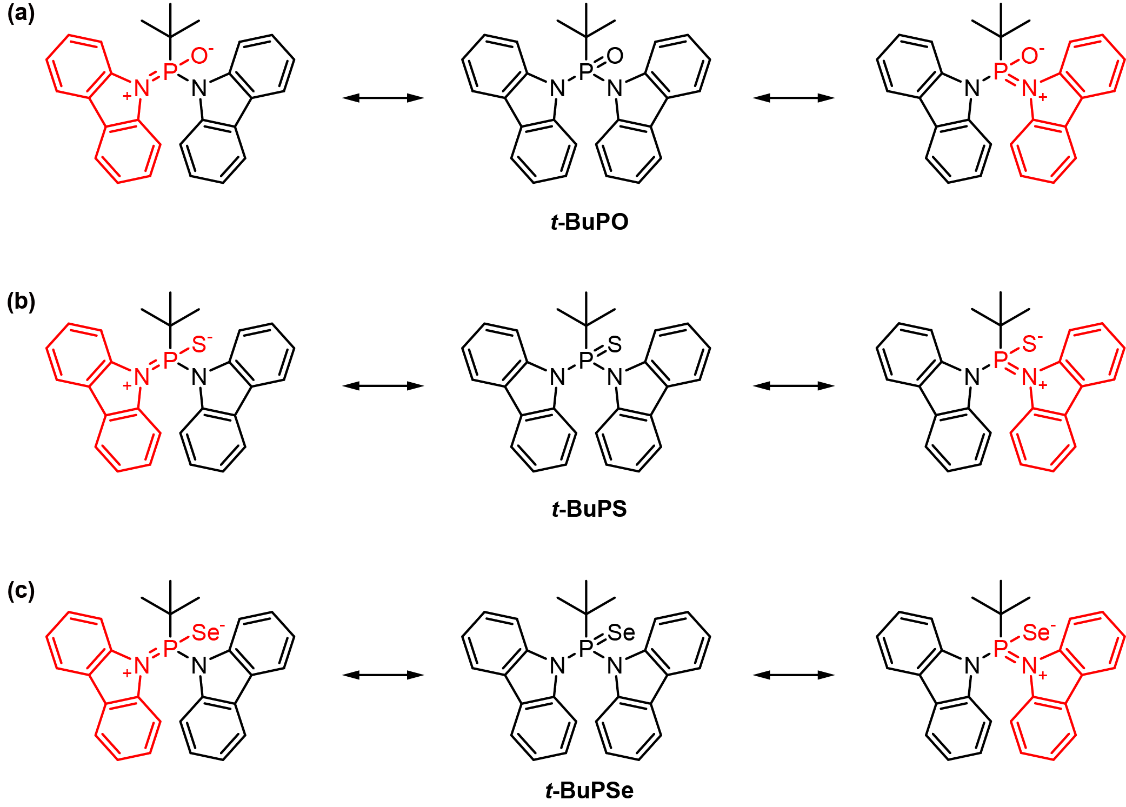


**Scheme S2.** Main resonance structures of (a) ***t*-BuPO**, (b) ***t*-BuPS**, and (c) ***t*-BuPSe**.

**5. Computational methods**

Density functional theoretical (DFT) calculations were performed on Gaussian 09 program. The highest occupied molecular orbital (HOMO), the lowest unoccupied molecular orbital (LUMO) energy levels, frontier molecular orbital distributions, and spin density distributions were predicted by B3LYP/6-31G(d) based on the optimized ground state (S0) geometries, since B3LYP/6-31G(d) is good in predicting the molecular energy levels of organic optoelectronic molecules.^[4]^ Natural bond orbital (NBO) analysis was performed to study the charge distribution of the D-r-D molecules. The distribution of the injected charge (*δ*_C_) on the carbazole and *tert*-butylphosphine oxide (sulfide or selenide) (BPX, X=O, S, Se) was calculated by **Equations S1** and **S2** when adding a negative (-1) charge on the molecule:

Carbazole (*δ*_C_) = Carbazole (-1) - Carbazole (0) (S1)

BPX (*δ*_C_) = BPX (-1) - BPX (0) (S2)

And, when a positive (+1) charge is injected, its charge distribution can be figured out by **Equations S3** and **S4**:

Carbazole (*δ*_C_) = Carbazole (+1) - Carbazole (0) (S3)

BPX (*δ*_C_) = BPX (+1) - BPX (0) (S4)

**Table S2.** The selected bond lengths of D-r-D molecules from single crystal structures.

| **Compound** | **N-P [Å]** | **P=X [Å]^a)^** | **P-C [Å]** | **N-C [Å]** |
| --- | --- | --- | --- | --- |
| ***t*-BuPO** | 1.700/1.692 | 1.463 | 1.817 | 1.430 |
| ***t*-BuPS** | 1.710/1.709 | 1.935 | 1.845 | 1.433 |
| ***t*-BuPSe** | 1.713/1.687 | 2.090 | 1.846 | 1.408 |

^a)^ X is O in ***t*-BuPO**; S in ***t*-BuPS**; Se in ***t*-BuPSe**.

**Table S3.** The selected bond lengths and charge distributions of D-r-D molecules.

| **Compound** | **N-P [Å]** | | |  | **Carbazoles** **[*δ*_C_]** | |  | **BPX [*δ*_C_]^a)^** | |
| --- | --- | --- | --- | --- | --- | --- | --- | --- | --- |
|  | -1 | 0 | +1 |  | -1 | +1 |  | -1 | +1 |
| ***t*-BuPO** | 1.731/1.715 | 1.735/1.734 | 1.782/1.743 |  | -0.577/-0.372 | 0.382/0.562 |  | -0.046 | 0.057 |
| ***t*-BuPS** | 1.740/1.726 | 1.751/1.746 | 1.778/1.764 |  | -0.517/-0.428 | 0.494/0.380 |  | -0.057 | 0.125 |
| ***t*-BuPSe** | 1.743/1.721 | 1.744/1.737 | 1.753/1.738 |  | -0.544/-0.384 | 0.437/0.293 |  | -0.065 | 0.271 |

^a)^ X is O in ***t*-BuPO**; S in ***t*-BuPS**; Se in ***t*-BuPSe**.

Bond order analysis, resonance variation energy, localized orbital locator profile, reduced density gradient and reorganization energy

Fuzzy bond order analysis embedded in Multiwfn was used to study the bond order of the resonance structures based on the optimized molecular structures at the ground state (S_0_). NBO 6.0 program was used to perform NBO energetic analysis with B3LYP/6-31G(d) method based on the single crystal structures. The energy of the resonance variation (*E*_RV_) was estimated using the energy difference of idealized natural Lewis structure of N-P=X and N^+^-P=X^-^ by deleting all Fock matrix elements between Lewis NBOs and the vicinal non-Lewis NBOs. Localized orbital locator (LOL) profiles of these D-r-D molecules were calculated at the B3LYP/6-31G(d) level by using Multiwfn.^[6,7]^ The nonbonding covalent interaction (NCI) analysis were adopted to investigate the intramolecular interactions using Multiwfn version 3.3 software based on the dimer structures from the single crystal structures. NCI isosurface plots were performed with color scaling that the blue color represents attractive interactions, while red color represents repulsive interactions. All the plotted isosurfaces were demonstrated with reduced density gradient (RDG) of 0.5 and -0.5 < sign(*λ*_2_)ρ < 0.5, where sign(*λ*_2_) means the sign of the second largest eigenvalue of Hessian and ρ represents the electron density.

Reorganization energies of hole (*λ*_h_) and electron (*λ*_e_) were calculated by using B3LYP/6-31G(d) method to assess the charge (hole and electron) mobility of D-r-D molecules based on the incoherent hopping model. This model assumes a charge transport process between two adjacent reactions M^±^ + M→M + M^±^, where M is the neutral molecule interacting with the neighboring cationic or anionic molecule (M^±^) to transfer the charge between them. Theoretically, *λ*_h_ and *λ*_e_ can be calculated by the following equations:^[8]^

$\text{λ}_{\text{h}}\text{=}\text{λ}_{\text{+}}\text{+}\text{λ}_{\text{1}}$ (S5)

$\text{λ}_{\text{e}}\text{=}\text{λ}_{\text{-}}\text{+}\text{λ}_{\text{2}}$ (S6)

$\text{λ}_{\text{+}}\text{=}\text{E}^{\text{+}}\text{(M)-}\text{E}^{\text{+}}\text{(}\text{M}^{\text{+}}\text{)}$ (S7)

$\text{λ}_{\text{1}}\text{=}\text{E}\text{(}\text{M}^{\text{+}}\text{)-}\text{E}\text{(M)}$ (S8)

$\text{λ}_{\text{-}}\text{=}\text{E}^{\text{-}}\text{(M)-}\text{E}^{\text{-}}\left( \text{M}^{\text{-}} \right)$ (S9)

$\text{λ}_{\text{2}}\text{=}\text{E}\text{(}\text{M}^{\text{-}}\text{)-}\text{E}\text{(M)}$ (S10)

where $\text{λ}_{\text{±}}$ are the relaxation energy of a neutral molecule (M) that captured a hole or electron going toward the optimum geometry on the potential energy surface of cation (M^+^) or anion (M^−^); *λ*_1_ and *λ*_2_ are the relaxation energy from M^+^ or M^−^ extracting a hole or electron going toward the M optimum geometry on the potential energy surface of M; *E*^+^(M) and *E*^+^(M^+^) are the total energies of the cation species under the optimum geometry of M and M^+^, respectively; *E*(M) and *E*(M^+^) represent the total energies of the neutral molecule under the optimum geometry of M and M^+^, respectively; *E^-^*(M) and *E^-^*(M*^-^*) are the total energies of the anionic species under the optimum geometry of M and M*^-^*, respectively.

**Table S4.** Fuzzy bond order analysis of the D-r-D molecules.

| **Compound** | **Bond order** | | |
| --- | --- | --- | --- |
|  | N-P | P=X^a)^ | C-P |
| ***t*-BuPO** | 1.19/1.18 | 2.12 | 0.96 |
| ***t*-BuPS** | 1.18/1.17 | 1.83 | 0.94 |
| ***t*-BuPSe** | 1.18/1.18 | 1.74 | 0.93 |

^a)^ X is O in ***t*-BuPO**, S in ***t*-BuPS**, Se in ***t*-BuPSe**.

**Table S5.** Theoretical activation energy of the resonance variation (*E*_RV_) between N-P=X and N^+^-P=X^-^ resonance structures (X=O, S and Se)

| **Compound** | **Structure** | **Energy [a.u.]** | | ***E*_RV_ [eV]** | |
| --- | --- | --- | --- | --- | --- |
| ***t*-BuPO** | N^+^=P-O^-^ | | -1596.786855 | | 2.05 |
|  | N-P=O | | -1596.862169 | |  |
|  | N^+^=P-O^-^ | | -1596.80178 | | 1.64 |
| ***t*-BuPS** | N^+^=P-S^-^ | | -1919.432404 | | 0.97 |
|  | N-P=S | | -1919.468022 | |  |
|  | N^+^=P-S^-^ | | -1919.429797 | | 1.04 |
| ***t*-BuPSe** | N^+^=P-Se^-^ | | -3919.510706 | | 0.67 |
|  | N-P= Se | | -3919.535344 | |  |
|  | N^+^=P-Se^-^ | | -3919.506384 | | 0.79 |


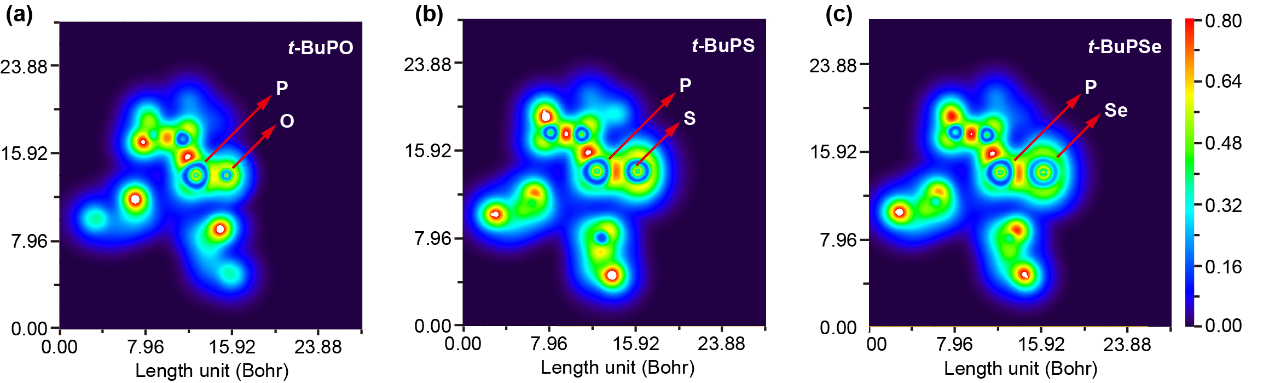


**Figure S12.** Localized orbital locator (LOL) profiles of (a) ***t*-BuPO**, (b) ***t*-BuPS** and (c) ***t*-BuPSe** using Multiwfn.

**
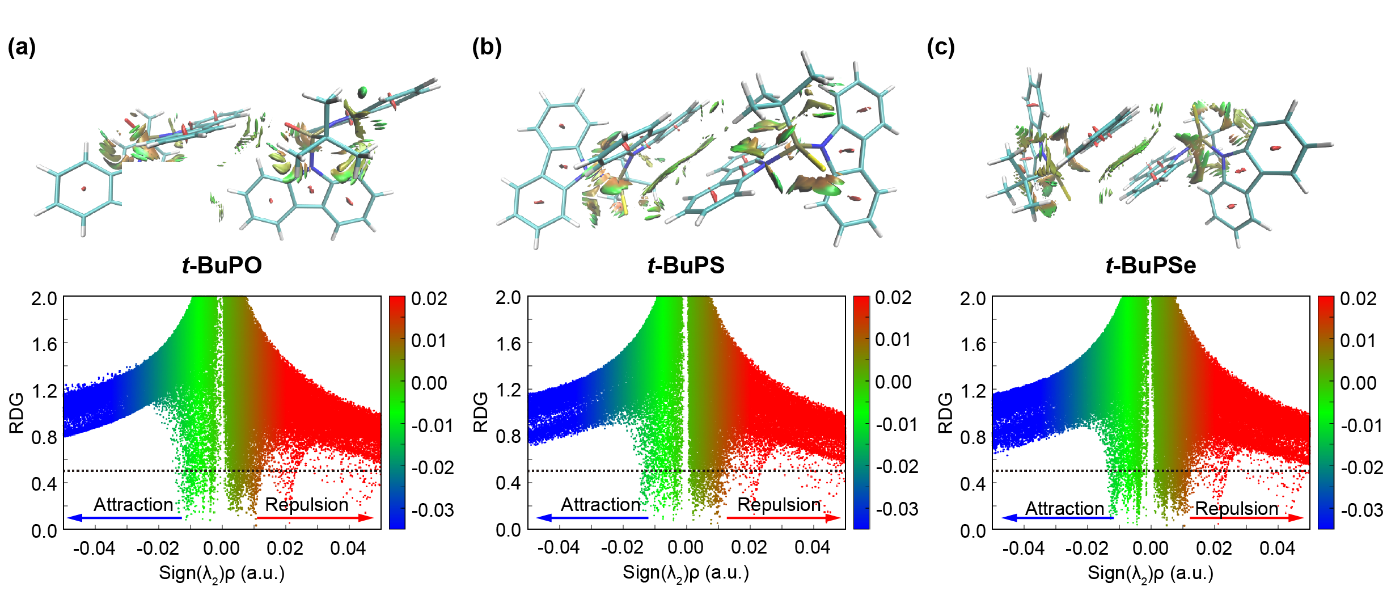
**

**Figure S13.** Reduced density gradient (RDG) versus sign(λ_2_)ρ with the view of the RDG isosurface of (a) ***t*-BuPO**, (b) ***t*-BuPS** and (c) ***t*-BuPSe** dimers. Positive charges are in red, while negative charges are in blue.

**6. Optical Properties**

Ultraviolet-visible (UV-Vis) spectra were recorded on a JASCO V-750 spectrophotometer, while fluorescence spectra were obtained on an Edinburgh FLS920 fluorescence spectrophotometer with a Xenon lamp as light source. The phosphorescence spectra of the compounds in CH_2_Cl_2_ were measured using a time resolved Edinburgh FLS920 fluorescence spectrophotometer at 77 K with a 5 ms delay time after the excitation (*λ*=290 nm) using a microsecond flash lamp.


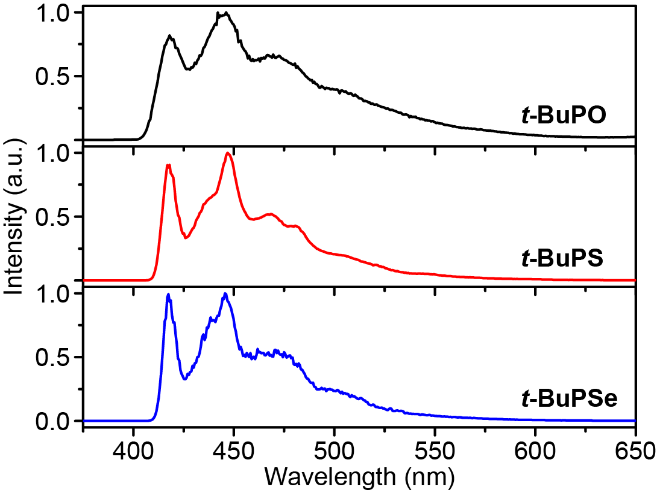


**Figure S14.** Phosphorescence spectra of the D-r-D molecules at 77 K in CH_2_Cl_2_ with a delay of 5 ms after the 290 nm excitation.


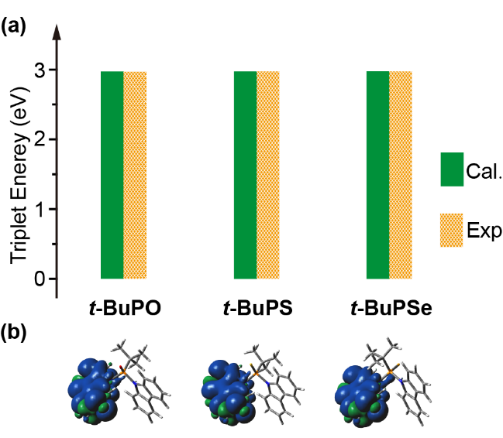


**Figure S15.** (a) Experimental and DFT calculated results of triplet energies and (b) spin density distributions of the D-r-D molecules.

**
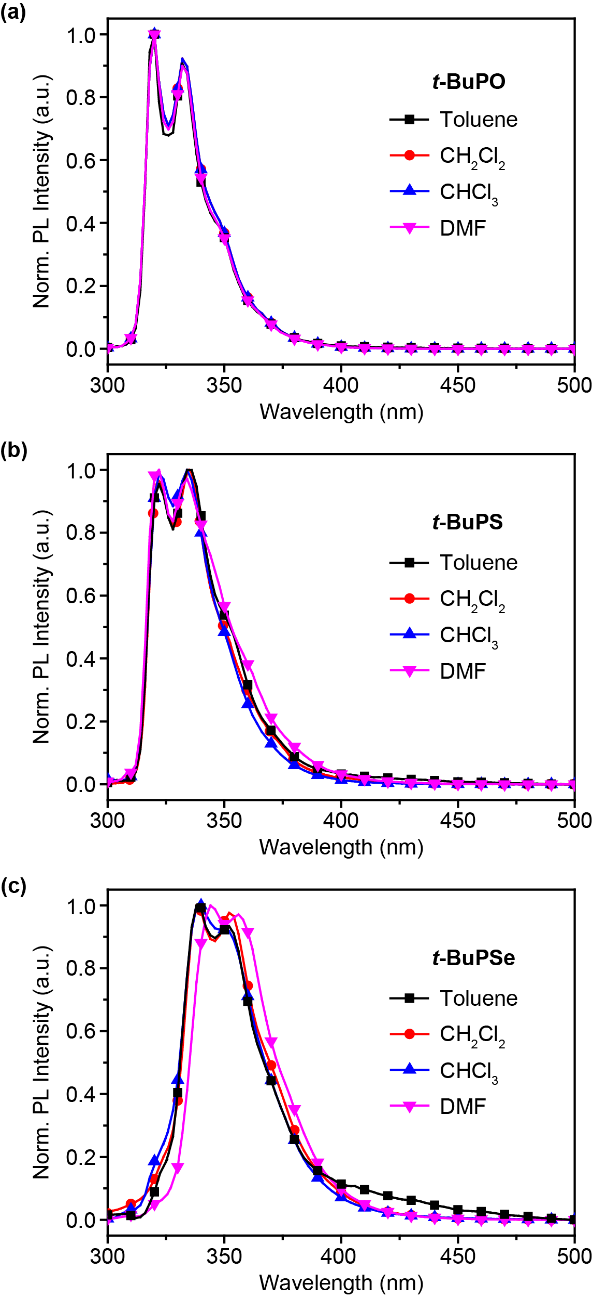
**

**Figure S16.** PL spectra of (a) ***t*-BuPO**, (b) ***t*-BuPS** and (c) ***t*-BuPSe** in different solvents with different polarities. The concentration of the emitters is ~10^-5^ mol L^-1^ and the excitation wavelength is at 290 nm.

**Table S6.** Photophysical and electrochemical properties of the D-r-D molecules.

| Comp. | *T*_d_/*T*_m_/*T*_g_  [°C] | *λ*_abs_ [nm] | | *E*_g_^a)^  [eV] | *λ*_em_ [nm] | | *E*_T_^b)^ [eV] | CV [eV] | |
| --- | --- | --- | --- | --- | --- | --- | --- | --- | --- |
|  |  | CH_2_Cl_2_ | Film |  | CH_2_Cl_2_ | Film |  | HOMO | LUMO^c^ |
| ***t*-BuPO** | 296.0/276.8/- | 287(304, 315) | 290(306, 318) | 3.85 | 320, 333 | 334 | 2.97 | -6.15 | -2.30 |
| ***t*-BuPS** | 255.0/245.0/- | 287(305, 316) | 291(307, 319) | 3.84 | 322, 335 | 362 | 2.97 | -6.16 | -2.32 |
| ***t*-BuPSe** | 235.0/230.2/145.0 | 288(305, 317) | 292(307, 319) | 3.82 | 338, 353 | 356 | 2.97 | -6.14 | -2.32 |
| **Carbazole** | -/244.8/- | 292(320, 333) | - | 3.65 | 341, 354 | - | 3.02 | -5.88 | -2.23 |

^a)^ Optical band gap (*E*_g_) calculated by the absorption edge technique in CH_2_Cl_2_; ^b)^ Triplet energy (*E*_T_) measured from phosphorescent spectrum at 77 K with a delay time of 5 ms; ^c)^ LUMO energy level estimated by adding *E*_g_ to the HOMO energy level.

**7. Electrochemical Properties**

Cyclic voltammetry (CV) measurements were performed at room temperature on a CHI660E system in a typical there-electrode cell with a working electrode (glass carbon), a reference electrode (Ag/Ag^+^, referenced against ferrocene/ferrocenium (FOC), and a counter electrode (Pt wire) in acetonitrile solution of tetrabutylammonium hexafluorophosphate **(**Bu_4_NPF_6_) (0.1 M) at a sweeping rate of 100 mV s^-1^. HOMO energy levels (*E*_HOMO_) of the materials were estimated based on the reference energy level of ferrocene (4.8 eV below the vacuum) according to **Equation S11**:

$E_{HOMO}=-\left[E_{onset}^{Ox}-E_{(Fc/Fc^{+})} +4.8\right] eV$ (S11)

where *E_(Fc/Fc_^+^_)_* is the onset potential of oxidative wave of ferrocene (Fc) *vs* Ag/Ag^+^ and $E_{\mathrm{onset}}^{\mathrm{Ox}}$ is the onset potential of the oxidation wave of the materials deposited as thin films on the surface of the working electrode. LUMO energy level (*E*_LUMO_) was estimated by adding the optical band-gap (*E*_g_) to the corresponding HOMO energy level as in **Equation S12**:

$E_{LUMO}=\left[E_{HOMO}+E_{g}\right]eV$ (S12)


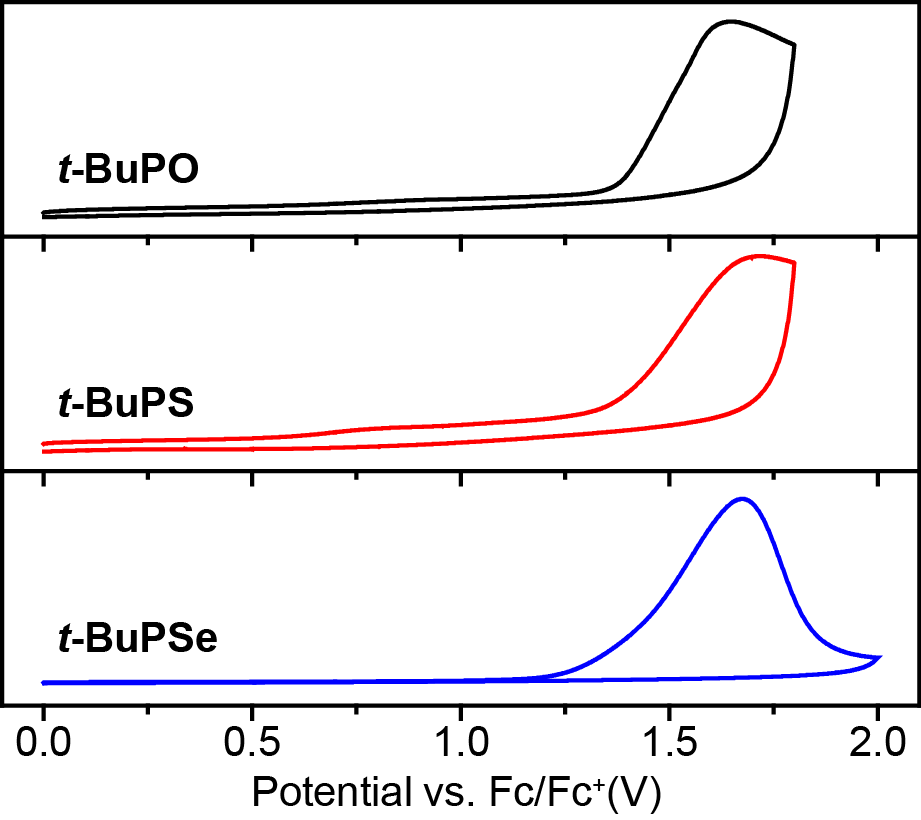


**Figure S17.** Cyclic voltammograms of the D-r-D molecules in thin solid films.

**8. Devices fabrication and measurements**

Single carrier transporting device. The charge transport properties were experimentally investigated by single-carrier charge transporting devices, under the devices structures of ITO/ PEDOT:PSS (30 nm)/ 1,3,5-Triazo-2,4,6-triphosphorine- 2,2,4,4,6,6-tetrachloride (TAPC) (20 nm)/ N, N′-dicarbazolyl-3,5-benzene (mCP) (8 nm)/ host (22 nm)/ mCP (8 nm)/ TAPC (20 nm)/ Al for hole only device and ITO/ LiF (1 nm)/ 1,3,5-tri(m-pyrid-3-yl-phenyl) benzene (TmPyPB) (35 nm)/host (22 nm)/ TmPyPB (35 nm)/ LiF (1 nm)/Al for electron-only device, respectively.^[2]^ The current density (*J*) in these devices follows the field-dependent space charge limit current (SCLC) model of Mott-Gurney law:

$J\text{=}\frac{\text{9}}{\text{8}}\text{ε}\text{ε}_{\text{0}}\text{μ}_{\text{0}}\text{exp}\left( 0.89\beta\sqrt{\frac{V}{d}} \right)\frac{\text{V}^{\text{2}}}{\text{d}^{\text{3}}}$ (S13)

$\mu\text{=}\text{μ}_{\text{0}}\text{exp}\left( \beta\sqrt{\frac{V}{d}} \right)$ (S14)

where *V* is the electrical voltage, *d* is the film thickness, *ε*_0_ is the permittivity of free space (8.854*10^-12^ F m^-1^), *ε* is the dielectric constant of the film, *μ* is the mobility at an electronic field, *μ*_0_ is the zero-field mobility, and *β* is the field-activation factor.^[9]^

Vacuum-deposited electroluminescence devices. Iridium(III) [bis(4,6-difluorophenyl)- pyridinato-N,C2’] picolinate (FIrpic) was selected as the blue phosphorescent dopants for the phosphorescent organic light emitting diodes (PhOLEDs) using these D-r-D molecules as host materials. The device structure of blue PhOLEDs is ITO/PEDOT:PSS (30 nm)/ TAPC (20 nm)/ *m*CP (8 nm)/ host: 15 wt% FIrpic (22 nm)/ TmPyPB (35 nm)/LiF (1 nm)/Al (100 nm). In these devices, *m*CP acts exciton-blocking layer; TAPC and TmPyPB function as the hole-transporting layer (HTL) and electron-transporting layer (ETL), respectively; and, PEDOT: PSS and LiF were used as hole- and electron-injecting layers, respectively. In a general procedure, ITO-coated glass substrates were etched, patterned, and washed with detergent, deionized water, acetone, and ethanol in turn. After ultraviolet (UV)-ozone treating for 4 min, PEDOT: PSS was spin coated on the ITO substrate (30 nm) and dried at 120ºC in a vacuum oven for 15 min. The other organic layers were deposited by thermal evaporation under a pressure of 4×10^-4^ Pa in a rate of 0.1-0.2 nm s^-1^. The layer thickness and the deposition rate were monitored in situ by oscillating quartz thickness monitors. The devices without encapsulation were measured immediately after fabrication under ambient atmosphere at room temperature. Electroluminescent (EL) spectra of the devices were measured by a PR655 spectra scan spectrometer. The luminance-voltage and current–voltage characteristics were recorded using an optical power meter and a Keithley 2602 voltage current source. And the external quantum efficiency (EQE) was calculated according to Equation S15.

$EQE=\frac{\pi e\eta_{cd/A}\int\lambda p(\lambda)d\lambda}{hcK_{m}\int p(\lambda)\Phi(\lambda)d\lambda}$ (S15)

where η_cd / A_ is the current efficiency (cd A^-1^); *h* is the Planck constant; *c* is the speed of light in vacuum; *λ* is the wavelength (nm); *e* is the electron charge; *p*(λ) is relative electroluminescent intensity at each wavelength; *Φ*(λ) is the Commission International del’Eclairage chromaticity (CIE) standard photopic luminous efficiency function; and *K*_m_ is a constant of 683 lm/W.

Solution-processed electroluminescence devices. Based on iridium(III) bis(4',6'- difluorophenylpyridinato)tetrakis(1-pyrazolyl)borate (FIr6), solution-processed deep blue PhOLEDs using D-r-D molecules as host materials were investigated. The deep-blue PhOLEDs are fabricated with device structure of ITO/PEDOT: PSS (60 nm)/host: 20 wt% 10-(4-((4-(9H- carbazol- 9-yl)phenyl)sulfonyl)phenyl)-9, 9- dimethyl- 9,10- dihydroacridine (CzAcSF): 15 wt% FIr6 (40 nm)/ bis[2-(diphenylphosphino) phenyl]ether oxide (DPEPO) (10 nm)/TmPyPB (50 nm)/8-hydroxyquinolinolato-lithium (Liq) (1 nm)/Al (100 nm). Similarly, TmPyPB was used as the electron-transporting layer (ETL); DPEPO acts as exciton-blocking layer; CzAcSF is the assistant host; and PEDOT: PSS and Liq are functioned as hole- and electron-injecting layers, respectively. The patterned ITO glass substrates were ultrasonically cleaned with detergent, alcohol, acetone, and deionized water for 30 min respectively, and then dried at 120°C in a vacuum oven for more than one hour. After ultraviolet (UV)-ozone treating for 15 min, a 60 nm PEDOT: PSS was spin coated on the ITO substrate and dried at 120°C in a vacuum oven for 15 min. Then, the emissive layers (EMLs) containing FIr6, D-r-D host molecules, and CzAcSF were spin-coated on the top of PEDOT: PSS from chlorobenzene and annealed using a hot plate at 80°C for 20 min to remove residual solvents. After that, the samples were transferred to a thermal evaporator chamber. DPEPO (10 nm), TmPyPB (50 nm), Liq (1 nm), and Al (100 nm) were deposited subsequently by thermal evaporation under a pressure of 5×10^−4^ Pa. The thickness of these vacuum-deposited layers was monitored using a spectroscopic ellipsometry (α-SE, J.A. Wollam Co. Inc.). The devices without encapsulation were measured immediately after fabrication at room temperature under ambient atmosphere conditions. The luminance-current-voltage (L-*J*-V) characteristics of the devices were recorded by a combination of a Keithley source-meter (model 2602) and a calibrated luminance meter. Electroluminescence (EL) spectra were obtained using a spectra-scan PR735 spectrophotometer. The external quantum efficiency (EQE) was achieved according to Equation S15.

**Table S7.** B3LYP/6-31G(d) calculated reorganization energies (*λ*_h_ for hole, *λ*_e_ for electron) and carrier mobilities (*μ*_h_ for hole, *μ*_e_ for electron) of the D-r-D molecules.

| Compound | *λ*_h_ (eV) | *λ*_e_ (eV) | *μ*_h_ (cm^2^V^-1^s^-1^)^a)^ | *μ*_e_ (cm^2^V^-1^s^-1^)^a)^ |
| --- | --- | --- | --- | --- |
| ***t*-BuPO** | 0.285 | 0.171 | 1.73*10^-7^ | 2.82*10^-9^ |
| ***t*-BuPS** | 0.146 | 0.166 | 9.18*10^-6^ | 1.12*10^-7^ |
| ***t*-BuPSe** | 0.210 | 0.176 | 4.59*10^-7^ | 5.32*10^-7^ |

^a)^ at an electric field of 0.3 MV/cm.

**
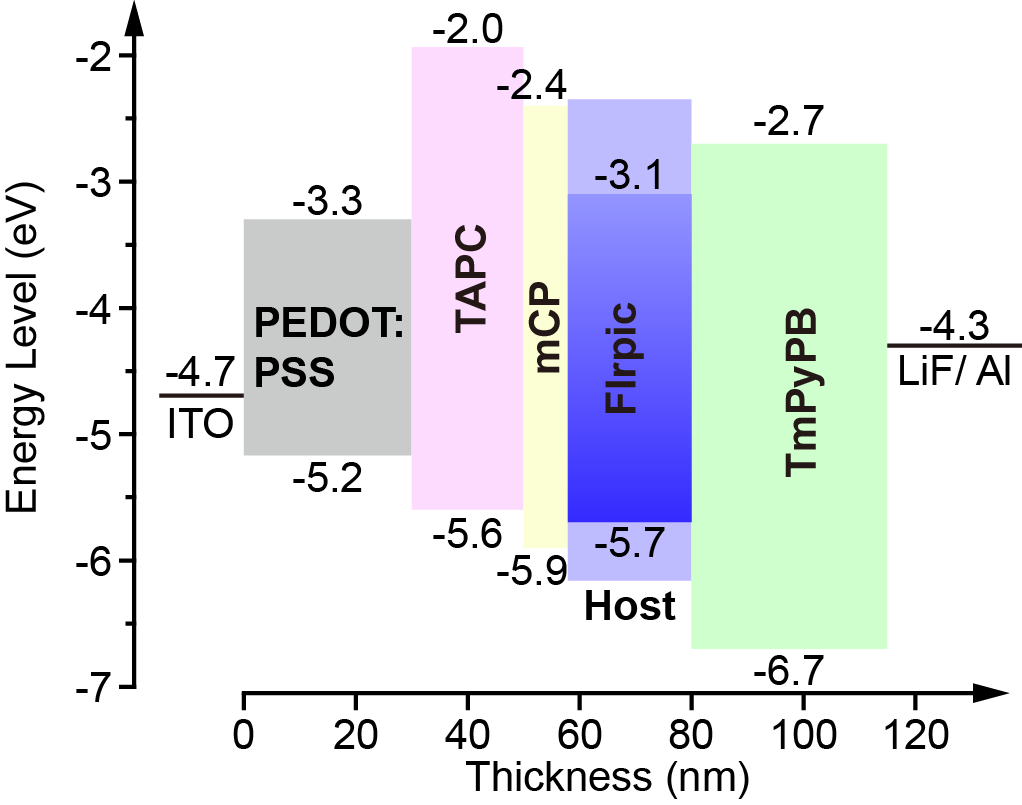
**

**Figure S18.** Device configuration and energy level diagram of the FIrpic-doped blue PhOLEDs hosted by ***t*-BuPO** and ***t*-BuPS**.


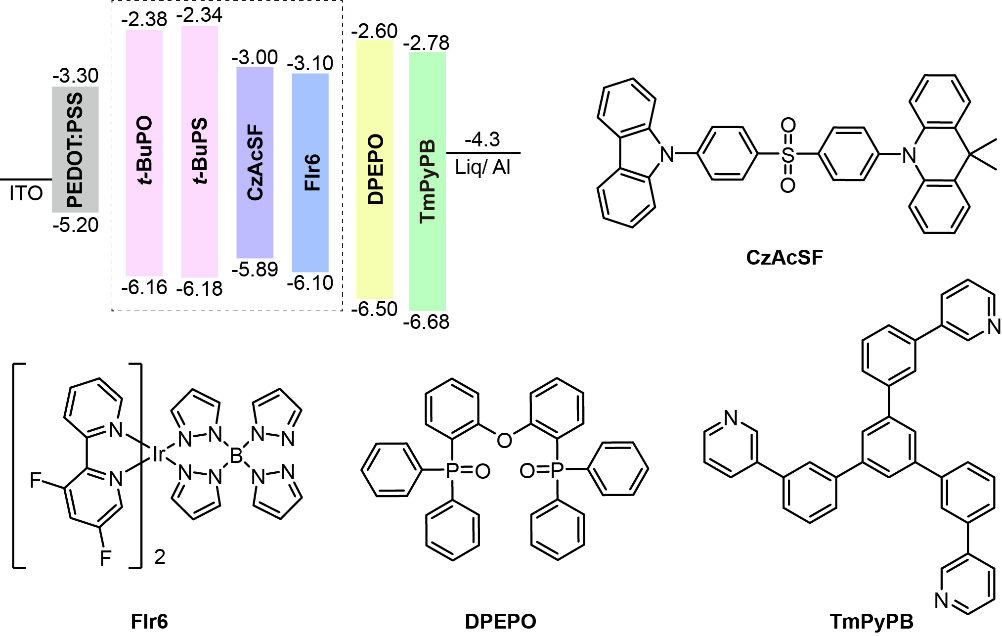


**Figure S19.** Device configuration and energy level diagram of the solution-processed FIr6-doped deep-blue PhOLEDs hosted by ***t*-BuPO** or ***t*-BuPS**.

**Table S8.** A brief summary of the reported device performance of the FIr6-based PhOLEDs using small molecular host materials.

| Host | Dopant Concentration | *V*_on_  [V] | EQE [%]^a)^ | CE [cd/A]^a)^ | PE [lm/W]^a)^ | Fabrication  Method | Reference |
| --- | --- | --- | --- | --- | --- | --- | --- |
| m-DCz-S | 10% | 2.8 | 25.1 | 49.8 | 50.9 | Vacuum | ^[10]^ |
| SPBI-TPA | 10% | 3.1 | 24.1 | 38.9 | - | Vacuum | ^[11]^ |
| DCDPOTZ | 10% | 2.8 | 22.9 | 36.6 | 41.8 | Vacuum | ^[12]^ |
| *p*-POSiTPA | 10% | 3.1 | 22.2 | 40.1 | 40.5 | Vacuum | ^[12]^ |
| POPCPA | 10% | 2.6 | 20.8 | 37.5 | 45.3 | Vacuum | ^[12]^ |
| m-Cz-S | 10% | 2.9 | 19.0 | 35.6 | 37.3 | Vacuum | ^[10]^ |
| POBPmDPA | 10% | 2.9 | 18.1 | 34.0 | 34.0 | Vacuum | ^[15]^ |
| S-DCz-Ph | 10% | 2.9 | 15.7 | 32.8 | 32.2 | Vacuum | ^[10]^ |
| CzCN-Ad | 10% | 2.9 | 14.2 | 26.5 | 26.8 | Vacuum | ^[16]^ |
| BTCC-36 | 10% | 5.0 | 6.8 | 11.5 | 4.9 | Spin-coating | ^[17]^ |
| *p*-TAZSiTPA | 10% | 5.3 | 6.3 | 12.5 | 6.2 | Spin-coating | ^[18]^ |
| POAPF | 10% | 2.5 | - | 11.3 | 13.7 | Vacuum | ^[19]^ |
| BTCC-27 | 10% | 5.1 | 5.8 | 9.8 | 4.3 | Spin-coating | ^[17]^ |
| BCC-36 | 10% | 4.9 | 4.2 | 7.4 | 3.1 | Spin-coating | ^[17]^ |
| BCC-27 | 10% | 5.0 | 4.1 | 6.8 | 2.8 | Spin-coating | ^[17]^ |
| ***t*-BuPO** | 15% | 4.7 | 12.1 | 22.9 | 11.5 | Spin-coating | This work |
| ***t*-BuPS** | 15% | 4.6 | 18.3 | 33.7 | 17.6 | Spin-coating | This work |

^a)^Maximum efficiency.


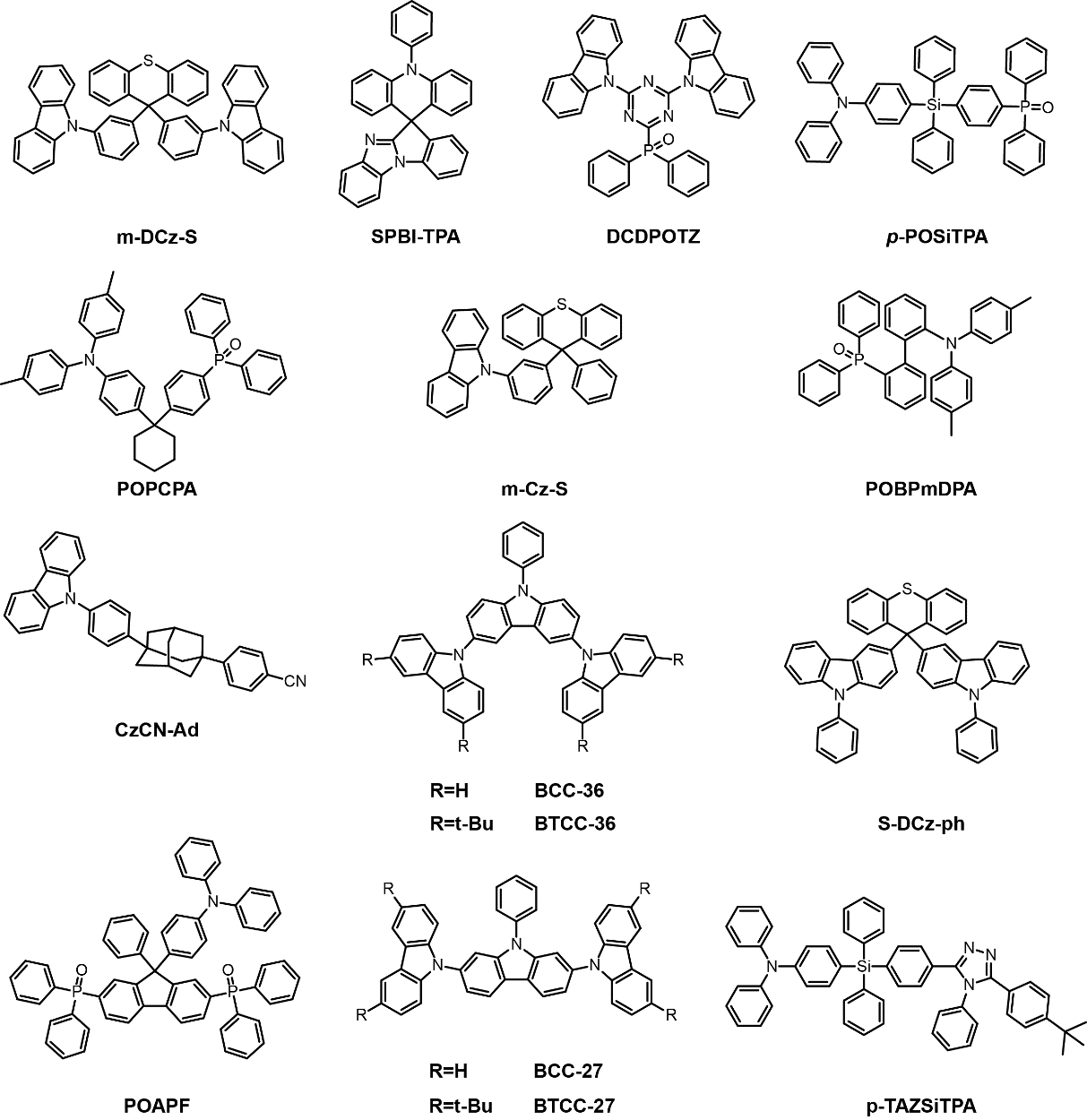


Scheme S3. Molecular structures of the host materials used in the FIr6-based PhOLEDs.

**References:**

[1] Y. Tao, J. Xiao, C. Zheng, Z. Zhang, M. Yan, R. Chen, X. Zhou, H. Li, Z. An, Z. Wang, H. Xu, W. Huang, *Angew. Chem. Int. Ed*. 2013, **52**, 10491.

[2] Y. Tao, L. Xu, Z. Zhang, R. Chen, H. Li, H. Xu, C. Zheng, W. Huang, *J. Am. Chem. Soc.* 2016, **138**, 9655.

[3] H. Jiang, L. Jia, Y. Li, S. Liu, R. Chen, L. Jin, J. Jin, C. Zheng, Q. Fan, W. Huang, *Chem. Commun.* 2018, **54**, 2926.

[4] F. Shojaie, *Comput. Theor. Chem.* 2017, **1114**, 55.

[5] Z. Mao, Z. Yang, Z. Fan, E. Ubba, W. Li, Y. Li, J. Zhao, Z. Yang, M. Aldred, Z. Chi, *Chem. Sci.* 2019, **10**, 179.

[6] T. Lu, F. Chen, *J. Comput. Chem.* 2012, **33**, 580.

[7] G. W. T. M. J. Frisch, H. B. Schlegel, G. E. Scuseria, M. A. Robb, J. R. Cheeseman, G. Scalmani, V. Barone, G. A. Petersson, H. Nakatsuji, X. Li, M. Caricato, A. Marenich, J. Bloino, B. G. Janesko, R. Gomperts, B. Mennucci, H. P. Hratchian, J. V. Ortiz, A. F. Izmaylov, J. L. Sonnenberg, D. Williams-Young, F. Ding, F. Lipparini, F. Egidi, J. Goings, B. Peng, A. Petrone, T. Henderson, D. Ranasinghe, V. G. Zakrzewski, J. Gao, N. Rega, G. Zheng, W. Liang, M. Hada, M. Ehara, K. Toyota, R. Fukuda, J. Hasegawa, M. Ishida, T. Nakajima, Y. Honda, O. Kitao, H. Nakai, T. Vreven, K. Throssell, J. A. Montgomery, Jr., J. E. Peralta, F. Ogliaro, M. Bearpark, J. J. Heyd, E. Brothers, K. N. Kudin, V. N. Staroverov, T. Keith, R. Kobayashi, J. Normand, K. Raghavachari, A. Rendell, J. C. Burant, S. S. Iyengar, J. Tomasi, M. Cossi, J. M. Millam, M. Klene, C. Adamo, R. Cammi, J. W. Ochterski, R. L. Martin, K. Morokuma, O. Farkas, J. B. Foresman, and D. J. Fox. *Journal*, 2016, Revision D.01.

[8] Y. Cheng, Y. Qi, Y. Tang, C. Zheng, Y. Wan, W. Huang, R. Chen, *J. Phys. Chem. Lett.* 2016, **7**, 3609.

[9] X. Yin, G. Xie, Y. Peng, B. Wang, T. Chen, S. Li, W. Zhang, L. Wang, C. Yang, *Adv. Funct. Mater.* 2017, **27**, 1700695.

[10] K. Gao, K. Liu, X. Li, X. Cai, D. Chen, Z. Xu, Z. He, B. Li, Z. Qiao, D. Chen, Y. Cao, S. Su, *J. Mater. Chem. C*. 2017, **5**, 10406.

[11] W. Chen, Y. Yuan, Z. Zhu, S. Ni, Z. Jiang, L. Liao, F. Wong, C. Lee, *Chem. Commun.* 2018, **54**, 4541.

[12] C. Li, X. Fan, C. Han, H. Xu, *J. Mater. Chem. C.* 2018, **6**, 6747.

[13] S. Gong, N. Sun, J. Luo, C. Zhong, D. Ma, J. Qin, C. Yang, *Adv. Funct. Mater.* 2014, **24**, 5710.

[14] S. Gong, Y. Chang, K. Wu, R. White, Z. Lu, D. Song, C. Yang, *Chem. Mater.* 2014, **26**, 1463.

[15] C. Fan, L. Zhu, T. Liu, B. Jiang, D. Ma, J. Qin, C. Yang, *Angew. Chem. Int. Ed.* 2014, **53**, 2147.

[16] Y. Gu, L. Zhu, Y. Li, L. Yu, K. Wu, T. Chen, M. Huang, F. Wang, S. Gong, D. Ma, J. Qin, C. Yang, *Chem-Eur. J.* 2015, **21**, 8250.

[17] W. Jiang, L. Duan, J. Qiao, G. Dong, D. Zhang, L. Wang, Y. Qiu, *J. Mater. Chem.* 2011, **21**, 4918.

[18] S. Gong, Q. Fu, Q. Wang, C. Yang, C. Zhong, J. Qin, D. Ma, *Adv. Mater.* 2011, **23**, 4956.

[19] Y. Yin, J. Yu, H. Cao, L. Zhang, H. Sun, W. Xie, *Sci. Rep-UK.* 2015, **4**, 6754.
