# Supplementary material for: Constructing Donor-Resonance-Donor Molecules for Acceptor-Free Bipolar Organic Semiconductors
Source: Research (Wash D C). 2021 Feb 5;2021:9525802. doi: 10.34133/2021/9525802 (PMC11014465; doi:10.34133/2021/9525802)
Supplement: Supplementary Materials — Figure S1: 1H NMR spectrum of t-BuPO in DMSO-d6. Figure S2: 13C NMR spectrum of t-BuPO in CDCl3. Figure S3: HRMS spectrum of t-BuPO. Figure S4: 1H NMR spectrum of t-BuPS in DMSO-d6. Figure S5: 13C NMR spectrum of t-BuPS in CDCl3. Figure S6: HRMS spectrum of t-BuPS. Figure S7: 1H NMR spectrum of t-BuPSe in DMSO-d6. Figure S8: 13C NMR spectrum of t-BuPSe in CDCl3. Figure S9: free volume region and fractional free volume (Vf) in the single crystal cells of (a) t-BuPO, (b) t-BuPS, and (c) t-BuPSe. Figure S10: (a) TGA and (b) DSC curves of the D-r-D molecules. Figure S11: AFM height images of the spin-coated thin films of (a) t-BuPO, (b) t-BuPS, and (c) t-BuPSe on ITO/PEDOT: PSS surface. Figure S12: localized orbital locator (LOL) profiles of (a) t-BuPO, (b) t-BuPS, and (c) t-BuPSe using Multiwfn. Figure S13: reduced density gradient (RDG) versus sign (λ2)ρ with the view of the RDG isosurface of (a) t-BuPO, (b) t-BuPS, and (c) t-BuPSe dimers. Positive charges are in red, while negative charges are in blue. Figure S14: phosphorescence spectra of the D-r-D molecules at 77 K in CH2Cl2 with a delay of 5 ms after the 290 nm excitation. Figure S15: (a) Experimental and DFT calculated results of triplet energies and (b) spin density distributions of the D-r-D molecules. Figure S16: PL spectra of (a) t-BuPO, (b) t-BuPS, and (c) t-BuPSe in different solvents with different polarities. The concentration of the emitters is ~10-5 mol L-1, and the excitation wavelength is at 290 nm. Figure S17: cyclic voltammograms of the D-r-D molecules in thin solid films. Figure S18: device configuration and energy level diagram of the FIrpic-doped blue PhOLEDs hosted by t-BuPO and t-BuPS. Figure S19: device configuration and energy level diagram of the solution-processed FIr6-doped deep-blue PhOLEDs hosted by t-BuPO or t-BuPS. Scheme S1: synthetic route of the D-r-D molecules: (i) n-BuLi, THF, t-BuPCl2, -78°C; (ii) 30% H2O2, CH2Cl2, room temperature (rt); (iii) sulfur, CH2Cl2, rt; (iv) selenium, [file 9525802.f1.zip › Supplementary Materials/t-BuPSe-checkCIF_PLATON report.pdf]

No syntax errors found.
Please wait while processing ....

CIF dictionary
Interpreting this report

Datablock: 1

Bond precision: C-C = 0.0145 A Wavelength=0.71073

Cell:	a=15.381(2)	b=9.8830(15)	c=15.381(2)
	alpha=90	beta=99.2400	gamma=90

Temperature: 290 K

	Calculated	Reported
Volume	2307.7(6)	2307.7(6)
Space group	P 21/n	P 21/n
Hall group	-P 2yn	-P 2yn
Moiety formula	C28 H25 N2 P Se	?
Sum formula	C28 H25 N2 P Se	C28 H25 N2 P Se
Mr	499.43	499.43
Dx,g cm-3	1.438	1.437
Z	4	4
Mu (mm-1)	1.715	1.715
F000	1024.0	1024.0
F000'	1024.31	
h,k,lmax	18,11,18	18,11,18
Nref	4066	2846
Tmin,Tmax	0.787,0.842	
Tmin'	0.787	

Correction method= Not given

Data completeness= 0.700 Theta(max)= 24.996

R(reflections)= 0.1001(1910) wR2(reflections)= 0.2383(2846)

S = 1.289 Npar= 292

The following ALERTS were generated. Each ALERT has the format
test-name_ALERT_alert-type_alert-level.
Click on the hyperlinks for more details of the test.

Alert level B

SYMMS02_ALERT_1_B The unit-cell lengths a and c should not be equal for a monoclinic cell

```
  Cell      15.3810    9.8830   15.3810
  Angles    90.0000   99.2400   90.0000
```

PLAT911_ALERT_3_B Missing FCF Refl Between Thmin & STh/L= 0.595 1222 Report

---

Alert level C

```
PLAT018_ALERT_1_C _diffrn_measured_fraction_theta_max .NE. *_full        ! Check
PLAT052_ALERT_1_C Info on Absorption Correction Method    Not Given    Please Do !
PLAT088_ALERT_3_C Poor Data / Parameter Ratio ....................       9.75 Note
PLAT234_ALERT_4_C Large Hirshfeld Difference C7        --C8       .      0.18 Ang.
PLAT341_ALERT_3_C Low Bond Precision on  C-C Bonds ...............    0.01448 Ang.
PLAT906_ALERT_3_C Large K Value in the Analysis of Variance ......     20.079 Check
```

And 2 other PLAT906 Alerts

```
PLAT906_ALERT_3_C Large K Value in the Analysis of Variance ......      3.773 Check
PLAT906_ALERT_3_C Large K Value in the Analysis of Variance ......      2.643 Check

PLAT934_ALERT_3_C Number of (Iobs-Icalc)/Sigma(W) > 10 Outliers ..          1 Check
```

Alert level G

```
PLAT145_ALERT_4_G s.u. on beta      Small or Missing .............     0.0000 Degree
PLAT883_ALERT_1_G No Info/Value for _atom_sites_solution_primary .    Please Do !
PLAT909_ALERT_3_G Percentage of I>2sig(I) Data at Theta(Max) Still        47% Note
PLAT941_ALERT_3_G Average HKL Measurement Multiplicity ...........        3.1 Low
PLAT965_ALERT_2_G The SHELXL WEIGHT Optimisation has not Converged    Please Check
PLAT978_ALERT_2_G Number C-C Bonds with Positive Residual Density.          0 Info
```

```
0 ALERT level A = Most likely a serious problem - resolve or explain
2 ALERT level B = A potentially serious problem, consider carefully
9 ALERT level C = Check. Ensure it is not caused by an omission or oversight
6 ALERT level G = General information/check it is not something unexpected

4 ALERT type 1 CIF construction/syntax error, inconsistent or missing data
2 ALERT type 2 Indicator that the structure model may be wrong or deficient
9 ALERT type 3 Indicator that the structure quality may be low
2 ALERT type 4 Improvement, methodology, query or suggestion
0 ALERT type 5 Informative message, check
```

It is advisable to attempt to resolve as many as possible of the alerts in all categories. Often the minor alerts point to easily fixed oversights, errors and omissions in your CIF or refinement strategy, so attention to these fine details can be worthwhile. In order to resolve some of the more serious problems it may be necessary to carry out additional measurements or structure refinements. However, the purpose of your study may justify the reported deviations and the more serious of these should normally be commented upon in the discussion or experimental section of a paper or in the "special_details" fields of the CIF. checkCIF was carefully designed to identify outliers and unusual parameters, but every test has its limitations and alerts that are not important in a particular case may appear. Conversely, the absence of alerts does not guarantee there are no aspects of the results needing attention. It is up to the individual to critically assess their own results and, if necessary, seek expert advice.

Publication of your CIF in IUCr journals

A basic structural check has been run on your CIF. These basic checks will be run on all CIFs submitted for publication in IUCr journals (*Acta Crystallographica*, *Journal of Applied Crystallography*, *Journal of Synchrotron Radiation*); however, if you intend to submit to *Acta Crystallographica Section C* or *E* or *IUCrData*, you should make sure that full publication checks are run on the final version of your CIF prior to submission.

Publication of your CIF in other journals

Please refer to the *Notes for Authors* of the relevant journal for any special instructions relating to CIF submission.

PLATON version of 18/09/2020; check.def file version of 20/08/2020

Datablock 1 - ellipsoid plot

Download CIF editor (publCIF) from the IUCr
Download CIF editor (enCIFer) from the CCDC
Test a new CIF entry
